# Overloaded Adeno-Associated Virus as a Novel Gene Therapeutic Tool for Otoferlin-Related Deafness

**DOI:** 10.3389/fnmol.2020.600051

**Published:** 2021-01-07

**Authors:** Vladan Rankovic, Christian Vogl, Nele M. Dörje, Iman Bahader, Carlos J. Duque-Afonso, Anupriya Thirumalai, Thomas Weber, Kathrin Kusch, Nicola Strenzke, Tobias Moser

**Affiliations:** ^1^Institute for Auditory Neuroscience and InnerEarLab, University Medical Center Göttingen, Göttingen, Germany; ^2^Restorative Cochlear Genomics Group, Auditory Neuroscience and Optogenetics Laboratory, German Primate Center, Göttingen, Germany; ^3^Presynaptogenesis and Intracellular Transport in Hair Cells Group, Institute for Auditory Neuroscience and InnerEarLab, University Medical Center Göttingen, Göttingen, Germany; ^4^Collaborative Research Center 889, University of Göttingen, Göttingen, Germany; ^5^Auditory Systems Physiology Group, Institute for Auditory Neuroscience and Department of Otolaryngology, University Medical Center Göttingen, Göttingen, Germany; ^6^Auditory Neuroscience Group, Max Planck Institute of Experimental Medicine, Göttingen, Germany; ^7^Multiscale Bioimaging Cluster of Excellence (MBExC), University of Göttingen, Göttingen, Germany; ^8^Synaptic Nanophysiology Group, Max Planck Institute of Biophysical Chemistry, Göttingen, Germany; ^9^Auditory Neuroscience and Optogenetics Laboratory, German Primate Center, Göttingen, Germany

**Keywords:** gene therapy and therapeutic delivery, deafness/hearing loss, AAV (adeno-associated virus), Auditory Neuroscience, otoferlin, preclinical, *in vivo*, organotypic culture model

## Abstract

Hearing impairment is the most common sensory disorder in humans. So far, rehabilitation of profoundly deaf subjects relies on direct stimulation of the auditory nerve through cochlear implants. However, in some forms of genetic hearing impairment, the organ of Corti is structurally intact and therapeutic replacement of the mutated gene could potentially restore near natural hearing. In the case of defects of the otoferlin gene (*OTOF*), such gene therapy is hindered by the size of the coding sequence (~6 kb) exceeding the cargo capacity (<5 kb) of the preferred viral vector, adeno-associated virus (AAV). Recently, a dual-AAV approach was used to partially restore hearing in deaf otoferlin knock-out (*Otof-*KO) mice. Here, we employed *in vitro* and *in vivo* approaches to assess the gene-therapeutic potential of naturally-occurring and newly-developed synthetic AAVs overloaded with the full-length *Otof* coding sequence. Upon early postnatal injection into the cochlea of *Otof-*KO mice, overloaded AAVs drove specific expression of otoferlin in ~30% of all IHCs, as demonstrated by immunofluorescence labeling and polymerase chain reaction. Recordings of auditory brainstem responses and a behavioral assay demonstrated partial restoration of hearing. Together, our results suggest that viral gene therapy of DFNB9—using a single overloaded AAV vector—is indeed feasible, reducing the complexity of gene transfer compared to dual-AAV approaches.

## Introduction

According to the World Health Organization, ~466 million people world-wide suffer from disabling hearing loss (WHO, 2019). In the majority of these cases, hearing impairment is caused by exposure to damaging noise levels as well as aging, likely with a contribution of a genetic predisposition. Defects of individual so-called “deafness genes” explain at least 50% of hereditary hearing impairment, which affects 1–2 in 1,000 newborns. Currently, more than 150 non-syndromic gene loci have been identified in humans (Willems, 2000; Bowl et al., 2017; Michalski and Petit, 2019). At present, no curative therapeutic approaches are available for these patients. Hence, hearing aids or cochlear implants represent the standard of rehabilitation, but fail to fully recover normal hearing function. While novel gene therapeutic approaches for this and other conditions are currently in development, these treatments are not yet clinically available. However, several recent preclinical studies demonstrate that AAV-mediated gene replacement can restore hearing in mouse models for various deafness genes (e.g., Akil et al., 2012, Askew et al., 2015; Jung et al., 2015; György et al., 2017; Pan et al., 2017; Dulon et al., 2018; Akil et al., 2019; Al-Moyed et al., 2019; reviewed in Kleinlogel et al., 2020).

Among the targets for future gene therapy is the *OTOF* gene, which encodes the multi-C2-domain protein otoferlin. *OTOF* mutations lead to autosomal recessive non-syndromic prelingual deafness DFNB9 (Yasunaga et al., 1999) or hearing impairment that can be temperature sensitive (Varga et al., 2006). The prevalence of *OTOF-*related deafness accounts for up to 5–8% of autosomal recessive non-syndromic hearing loss cases in some Western populations (Rodríguez-Ballesteros et al., 2008) and hence, resides within the top five of genetic hearing disorders that require therapeutic intervention (Angeli et al., 2012). Otoferlin is abundantly expressed in sensory inner hair cells (IHCs) of the cochlea and serves a key role in the final steps of synaptic vesicle fusion at IHC ribbon synapses with afferent spiral ganglion neurons (Roux et al., 2006). Here, several functions of otoferlin have been proposed [reviewed in Moser and Starr (2016), Moser et al. (2019)], such as a role in (i) presynaptic Ca^2+^-sensing to trigger vesicular exocytosis upon IHC depolarization (Roux et al., 2006; Johnson and Chapman, 2010; Michalski et al., 2017) and (ii) efficient vesicular priming and replenishment of synaptic vesicles to the release site to ensure indefatigable and temporally-precise neurotransmitter release during periods of prolonged stimulation (Pangrsic et al., 2010; Jung et al., 2015; Vogl et al., 2015, Strenzke et al., 2016; Vogl et al., 2016). Mouse mutants serve as a valuable model system to investigate synaptic disease mechanisms (Moser and Starr, 2016) and together with clinical data [reviewed in Santarelli et al. (2015), Moser and Starr (2016)] indicate that preservation of cochlear morphology and function make otoferlin-related auditory synaptopathy amenable to clinical gene therapy.

Medical need and permissive cochlear status have generated great interest in gene therapy approaches to otoferlin-related auditory synaptopathy. However, due to the large *OTOF* coding sequence of ~6 kb and the restricted packaging size of standard adeno-associated viral vectors (AAV; <4.7 kb), (Grieger and Samulski, 2005) that are commonly employed for gene therapeutic applications based on their favorable biosafety profile, the delivery of full-length *OTOF* remains challenging. Recently, two independent studies have overcome this problem by using a dual-AAV approach and could show partial rescue of hearing in *Otof-*KO mice (Akil et al., 2019; Al-Moyed et al., 2019). Yet, the complexity of this approach makes clinical translation more challenging than in cases where a single AAV serves as the molecular therapeutic [reviewed in Kleinlogel et al. (2020)].

Here, we aimed to establish a simplified strategy for delivering the coding sequence for full-length *Otof* (*fl-Otof*-CDS) to IHCs of *Otof-*KO mice using a single AAV “overloading” strategy (Allocca et al., 2008; Grose et al., 2012; Hirsch et al., 2013; Pryadkina et al., 2015). Conceptually, this approach is based on attempting to package a CDS, which theoretically exceeds the predicted maximum insert capacity of the employed AAV—in this particular case, the *fl-Otof*-CDS (~6 kb) into a standard AAV (maximum capacity: 4.7 kb)—thereby technically “overloading” the virus. In fact, to establish the proof-of-principle for this approach for future cochlear gene therapy, we packaged the *fl-Otof*-CDS into not only one, but rather several naturally-occurring, as well as more recently developed and highly potent synthetic AAV serotypes. We then tested the potential of these overloaded AAVs for expressing functional otoferlin in IHCs of the mouse organ of Corti in organotypic cultures as well as in living mice upon early postnatal AAV injection. We show expression of *fl-Otof* in IHCs of *Otof-*KO mice that went along with partial hearing restoration, thereby demonstrating that single AAV overloading provides a feasible gene-therapeutic strategy for otoferlin-related auditory synaptopathy. Moreover, our data indicate that successful gene transfer by overloaded AAVs can be achieved independent of the employed AAV capsid composition.

## Materials and Methods

### Molecular Cloning

Full-length mouse otoferlin (*fl-Otof*) was subcloned from a previously generated cDNA clone (pcDNA3-mOtof-IRES-EGFP) into a pAAV vector. The cloning procedure was done by in-fusion cloning according to manufacturer instructions (TaKaRa/Clontech, In-Fusion HD Cloning kit). Owing to the size of *fl-Otof* we amplified three overlapping otoferlin fragments which complement *fl-Otof* by high fidelity PCR using the HiFi-PCR-premix provided by the kit and fused into the linearized pAAV target vector to generate pAAV_*fl-Otof*. The corresponding vector map of pAAV_*fl-Otof* with ubiquitous CMV-β-Actin (CMV/hbA) hybrid promoter is shown in Supplementary Figure 1. Due to the lack of suitable restriction sites within the otoferlin coding region and the substantial length of the *fl-Otof* cDNA we refrained from using traditional T4 ligase-based cloning techniques. Instead, we applied in fusion cloning strategy to generate a *fl-Otof* plasmid. To this end we linearized the target vector for virus expression (pAAV) by digestion with the restriction enzymes *Nhe*I and *Hind*III (Fermentas). We amplified three otoferlin fragments complementing *fl-Otof* [fragment A (primer pair A_F2/R1), fragment B (B_F1/R1), and fragment C (C_F1/R2)], which contained overlapping regions with each other and with the linearized target vector (see Appendix A1). This step required fragment and primer optimization [e.g., by determining optimal fragment sizes and ratios and by using “split overlaps” to reduce primer lengths (primers see Appendix A1.1 and A1.2)]. Thus, the linearized vector and three fragments were simultaneously fused together using the TaKaRa/Clontech *In-Fusion HD* Cloning kit, following manufacturer's instructions. This approach yielded the final *fl-Otof* viral vector (pAAV_*fl-Otof*) used for subsequent virus production (Supplementary Figure 1B). Target pAAV vector linearized by *Nhe*I/*Hind*III digest and PCR-amplified otoferlin fragments A, B, C using the primer pairs are shown in Supplementary Figure 1A and Appendix A1.2. Note that primer A_F2 contains a 15 bp overlap with the target vector at the 5'-end and encodes the START-codon for expression of otoferlin, while C_R2 encodes the STOP codon and a 15 bp overlap with the AAV vector at the 3'-end. Restriction enzyme digestions and Sanger sequencing (SeqLab, Germany) was employed to verify the correct *fl-Otof* insert. The length of the otoferlin coding region is 5,979 kb comprising the mouse transcript variant 1 which contains the alternative Exon5B (NCBI accession numbers NP_001093865.1, Appendix A4). This sequence shows 94% identity to the human otoferlin sequence (NP_001274418.1), as determined by using the BLASTP algorithm (NCBI). A protein alignment of mouse and human otoferlin is shown in the Appendix A4 [CLUSTAL O(1.2.4)]. The CDS size (5,979 kb) is beyond the packaging capacity of adeno-associated virus of 4.7 kb (according to (Grieger and Samulski, 2005). A truncated otoferlin, consisting only of the C2E and C2F including the transmembrane domain (TM)—named in this study as “miniOtoferlin” (Supplementary Figures 2, 3A)—was generated in a similar way. Briefly, two overlapping fragments covering the C-terminal part of *Otof* were amplified from the cDNA clone (pcDNA3-mOtof-IRES-EGFP) by high fidelity PCR using the HiFi-PCR-premix provided (TaKaRa/Clontech, In-Fusion HD Cloning kit) and overlapping primer pairs (G_F1/B_R1 and C_F1/R1 for fragments G and C2, respectively) (see Appendix A1). For linking to EGFP, both fragments were fused simultaneously into pEGFP-C2 linearized by SacI (Thermo Fisher Scientific) using the in-fusion reaction according to the manufacturer's instructions (TaKaRa/Clontech, In-Fusion HD Cloning kit). The resulting *EGFP-miniOtof* fusion construct was subcloned to the linearized pAAV vector. Insert and vector were restricted using NheI and HindIII (Thermo Fisher Scientific) and complementary ends were ligated using T4 ligase. This approach yielded the final *EGFP-miniOtof* viral vector (pAAV_*EGFP-miniOtof*) used for subsequent virus production. Restriction enzyme digestion and Sanger sequencing (SeqLab, Germany) was employed to verify the correct *EGFP-miniOtof* insert.

### Virus Production and Purification

AAVs were generated in HEK 293 T cells (ATCC) using polyethylenimine transfection (25,000 MW, Polysciences, USA) (Gray et al., 2011; Deverman et al., 2016). In brief, triple transfection of HEK 293 T cells was performed using pHelper plasmid (TaKaRa/Clontech), trans-plasmid providing viral capsid PHP.B (generous gift from Ben Deverman and Viviana Gradinaru, Caltech, USA), PHP.eB (gift from Viviana Gradinaru, Addgene plasmid # 103005; http://n2t.net/addgene:103005; RRID:Addgene_103005), Anc80L65AAP (gift from Luk Vandenberghe, Addgene plasmid # 92307; http://n2t.net/addgene:92307; RRID:Addgene_92307), AAV2/8 (gift from James M. Wilson (Addgene plasmid # 112864; http://n2t.net/addgene:112864; RRID:Addgene_112864), or AAV2/9 (Penn Vector Core, University of Pennsylvania) and cis-plasmid providing *fl-Otof* (see Supplementary Figure 1B). The cell line was regularly tested for mycoplasma. We harvested viral particles 72 h after transfection from the medium and 120 h after transfection from cells and the medium. Viral particles from the medium were precipitated with 40% polyethylene glycol 8000 (Acros Organics, Germany) in 500 mM NaCl for 2 h at 4°C and then after centrifugation at 4,000 g for 30 min combined with cell pellets for processing. The cell pellets were suspended in 500 mM NaCl, 40 mM Tris, 2.5 mM MgCl2, pH 8, and 100 U/mL of salt-activated nuclease (Arcticzymes, USA) at 37°C for 30 min. Afterwards, the cell lysates were clarified by centrifugation at 2,000 *g* for 10 min and then purified over iodixanol (Optiprep, Axis Shield, Norway) step gradients (15, 25, 40, and 60%) (Zolotukhin et al., 1999; Grieger et al., 2006) at 58,400 rpm for 2.25 h. Using this approach, empty AAV particles, cellular proteins, and deoxycholate residues are immobilized in the 40 and 25% iodixanol fraction and subsequently discarded. Finally, viruses were concentrated using Amicon filters (EMD, UFC910024) and formulated in sterile phosphate-buffered saline (PBS) supplemented with 0.001 % Pluronic F-68 (Gibco, USA) and titers measured using a AAV titration kit (TaKaRa/Clontech) according to manufacturer's instructions by determining the number of DNase I resistant vg using qPCR (StepOne, Applied Biosystems). Purity of produced viruses was routinely checked by silver staining (Pierce, Germany) after gel electrophoresis (Novex™ 4–12% Tris-Glycine, Thermo Fisher Scientific) according to manufacturer's instruction. The presence of viral capsid proteins was positively confirmed in all virus preparations. Viral stocks were kept at −80°C until the experimental day.

### Organotypic Culture Preparation and AAV Inoculation

Organ of Corti explant cultures were essentially prepared as previously described (Kroll et al., 2019). In brief, cochleae were harvested from p5 *Otof-*KO mice and organs of Corti of the apico-medial cochlear turn micro-dissected in modified HBSS [HBSS (Thermo Fisher), 10 mM HEPES (Thermo Fisher), 250 ng/ml fungizone (Thermo Fisher), and 10 μg/ml penicillin G (Sigma Aldrich)]. Finally, the explants were mounted on CellTak (BD Bioscience) -coated coverslips in serum-free DMEM/F12 medium [DMEM/F12, 1% N2 supplement (Thermo Fisher) and ampicillin (Sigma Aldrich)] and cultured overnight in a humidified incubator (37°C; 5% CO_2_). After 12 h, the coverslips were transferred to 24-well plates for AAV inoculation in growth medium (48 h; DMEM/F12, 1% N2 supplement, 1% ampicillin, 1% newborn calf serum [Thermo Fisher]), with the following AAV titers: AAV2/8: 3.2 × 10^12^ genome copies/ml; AAV2/9: 2.4 × 10^12^ genome copies/ml; Anc80L65: 6.0 × 10^12^ genome copies/ml; PHP.B: 1.3 × 10^12^ genome copies/ml; PHP.eB: 5.8 × 10^12^ genome copies/ml in 10 μl/well. Subsequently, the growth medium was exchanged 50:50 every 2 days prior to fixation and histological evaluation at DIV9-11.

### Postnatal AAV-Injection

Postnatal AAV-injection into the scala tympani of the left ear via the round window was performed at p5-7 essentially as described in Akil et al. (2012). In this study, we used PHP.B-derived viral capsids in combination with a (CMV/hbA) promoter to drive transgenic expression of *fl-Otof* in cochlear IHCs. Both female and male animals were used equally during the study. Briefly, around 1-1.5 μl of PHP.B_CMV/hbA-*fl-Otof* (1.3 × 10^12^ genome copies/ml) or PHP.eB_CMV/hbA-*fl-Otof* (5.0 × 10^12^ genome copies/ml), AAV2/9_CMV/hbA-*fl-Otof* (2.4 × 10^12^ genome copies/ml), Anc80L65_CMV/hbA-*fl-Otof* (6.0 × 10^12^ genome copies/ml), and PHP.eB_CMV/hbA_eGFP-mini-Otof (4.9 × 10^12^ genome copies/ml) were injected into the left ear *via* the round window. 4 weeks after injection, animals were used in auditory brainstem (ABR) recording experiments or Intellicage experiments. All experiments were done in compliance with the national animal care guidelines and were approved by the board for animal welfare of the University Medical Center Goettingen and the animal welfare office of the state of Lower Saxony (AZ33.9-42502-04-14/1391).

### Immunohistochemistry and Confocal Microscopy

Acutely dissected or organotypically-cultured cochlear explants were fixed in 4% formaldehyde in PBS on ice for 1 h. Strongly ossified cochleae of adult mice were decalcified in 10% EDTA solution (prepared in PBS) for 10–11 days prior to dissection. After extensive washing, permeabilization (30 min; 0.5% Triton X-100 in PBS) and a blocking step with a goat serum dilution buffer (GSDB; 1 h; 16% normal goat serum, 450 mM NaCl, 0.3% Triton X-100 and 20 mM phosphate buffer at pH 7.4), the following primary antibodies were applied in GSDB overnight at 4°C: mouse anti-otoferlin (Cat.-Nr. ab53233; Abcam) or rabbit anti-otoferlin (Vogl et al., 2015; Al-Moyed et al., 2019), chicken anti-calretinin (Cat.-Nr. 214 106; Synaptic Systems), guinea pig anti-parvalbumin (Cat.-Nr. 195 004; Synaptic Systems) and rabbit anti-Ribeye (Cat.-Nr. 192 103; Synaptic Systems). For visualization, secondary AlexaFluor-488,−568 and−647-conjugated antibodies (Cat.-Nr. A-11034, A-11011 or A-11075 and A-21236; Thermo Fisher Scientific) were applied the following day (1 h; room temperature). After mounting the specimen in Mowiol mounting medium, images were acquired in confocal mode on an Abberior Instruments Expert Line STED microscope controlled by Imspector software, with excitation lasers at 485, 561, and 640 nm and either a UPlanSApo 20x 0.85 NA or UPlanSApo 100x 1.4 NA oil immersion objectives. Image stacks were acquired with voxel sizes of 350 × 350 × 5,000 nm (20x) or 80 × 80 × 200 nm (100x).

Transduction efficiency was manually quantified either (i) across the entire organotypically-cultured organ explant, or (ii) along the entire length of the acutely dissected apical to medium cochlear turn (the average length of the analyzed fragments as measured from the cochlear apex: Group 1: 1966.4 ± 113.3 μm, *n* = 459 IHCs, *N* = 2; Group 2: 1988.4 ± 53.7 μm, *n* = 704 IHCs, *N* = 3;). Ribbon counts were established per IHC in the parvalbumin channel and cells were then assigned either to the transduced (i.e., otoferlin-positive) or non-transduced (i.e., otoferlin-negative) group, based on the observed otoferlin immunoreactivity.

Synapse counts were taken from clearly identifiable positively or adjacent negatively transduced IHCs. Data normality was established using a Shapiro-Wilk test. Normally distributed data sets were then analyzed using a one-way ANOVA followed by a *post-hoc* Tukey test to assess individual statistical significances.

### Chemical Tissue Clearing and Lightsheet Microscopy

The osseous labyrinth—i.e., cochlea and vestibular system—was dissected from the mouse temporal bone and fixed in 4% formaldehyde (diluted in PBS) for 1 h on ice. Subsequently, samples were decalcified in 10% EDTA for ~2 weeks at 4°C. For chemical tissue clearing and immunostaining protocol, we followed an adapted version of the iDISCO+ approach (Renier et al., 2016). Here, all washing and incubation steps were performed at 37°C with the samples continuously rotating. First, samples were incubated for 3 h in a permeabilization solution (0.5% Triton X-100 in PBS), which was followed by an overnight incubation in blocking solution (0.5% Triton X-100 + 10% v/v goat serum in PBS). Then, primary antibodies against Calretinin (Cat.-Nr. 214 106; Synaptic Systems) and Otoferlin (Cat.-Nr. 178 003; Synaptic Systems) were diluted in blocking solution and samples were incubated in the primary antibody solution for 48 h. After primary incubation, samples were washed in PBS for 24 h. The secondary antibodies goat anti-chicken 488 (Thermo Fisher, #A-11039) and goat anti-rabbit 647 (Thermo Fisher, #A-21244) were diluted in blocking solution and applied for 48 h. Finally, specimen were washed for 48 h and prepared for tissue clearing according to the iDISCO+ protocol (Renier et al., 2016).

Images were acquired with the LaVision UltraMicroscope II using the LaVision BioTech ImSpector Software. The numerical aperture was set to 0.148 which resulted in a sheet thickness of 5 μm. Stacks were acquired with a z step of 3 μm, a total zoom of 8x (2x objective plus 4x optic zoom) and an individual range for each cochlea that was covering all IHCs.

For analysis, images were imported into Imaris software (version 9.5.1, Oxford Instruments) to create a surface that contained all IHCs. This surface was as a mask to generate a new stack containing only the IHC row signal. Within this region of interest, we used a custom Matlab code that employed Greenwood's function (Greenwood, 1961) for tonotopic mapping of the IHC row, using parameters reported for the mouse cochlea in Müller et al. (2005). As an output of this code, we obtained 500 interpolated coordinates equally distributed along the tonotopic axis together with their corresponding frequency labels. By displaying the interpolated coordinates that most closely matched the frequency labels 4, 8, 12, 16, 24, and 32 kHz, we defined our frequency regions along the IHC row. IHCs within the respective frequency bands were finally counted in ImageJ/Fiji (Schneider et al., 2012) using the Cell Counter tool.

### RNA Isolation and Reverse Transcription

Total RNA was isolated from 8- to 10-weeks-old mice organs of Corti with the Invitrogen™ TRIzol™ Plus RNA Purification Kit (#12183555, Thermo Fisher Scientific) following the manufacturer's instructions. The SuperScript® IV First-Strand Synthesis System (#18091050, Thermo Fisher Scientific) served to synthesize cDNA according to manufacturer's instructions (see Appendix A2 for specific primer sets).

### Auditory Brainstem Responses and Otoacoustic Emissions

For recordings of ABR, mice were anesthetized with a combination of ketamine (125 mg/kg) and xylazine (2.5 mg/kg) i.p. The core temperature was maintained constant at 37°C using a heat blanket (Hugo Sachs Elektronik–Harvard Apparatus). For stimulus generation, presentation, and data acquisition, we used the TDT II System and BioSig software (Tucker Davis Technologies). Tone bursts (4/6/8/12/16/24/32 kHz, 10 ms plateau, 1 ms cos^2^ rise/fall) or clicks of 0.03 ms were presented at 40 Hz (tone bursts) or 20 Hz (clicks) in the free field ipsilaterally using a JBL 2402 speaker. The difference potential between vertex and mastoid subdermal needles was amplified 50,000 times, filtered (400–4,000 Hz), and sampled at a rate of 50 kHz for 20 ms, 1,300 times, to obtain two mean ABR traces for each sound intensity. Hearing thresholds were determined with 10 dB precision as the lowest stimulus intensity that evoked a reproducible response waveform in both traces by visual inspection. The injected ear of virus-injected Otof-KO was recorded first and then plugged with electrode gel and tissue paper to achieve a conductive hearing impairment of 30–40 dB (Pauli-Magnus et al., 2007). This limited acoustic cross-talk while recording from the non-injected contralateral ear, though some remaining activation *via* reflected sound waves and bone conduction is still possible. Tone burst thresholds exceeding the maximal loudspeaker output (80 dB for tone bursts, 100 dB for Clicks) were assigned a value of 90 and 110 dB, respectively, for the calculation of means and SEMs. Saturation of amplitude growth functions was defined as the intensity at which the amplitude (peak to trough of the entire waveform) reached 80% of the value obtained at 100 dB.

### Behavioral Tests of Hearing Function

In the Intellicage/Audiobox (New Behavior), operant conditioning of transponder-injected mice was performed 24/7 under computer control for 15 days. Mice visited the corner on average 60 times per day. In 90% of these visits, no stimulus was presented and the mice were allowed free access to water. In 10% of visits, 20 Hz click trains (5 clicks over 0.2 s, 0.3 s pause) at 100 dB were presented for the whole duration of the visit. Drink attempts during these visits were punished by a mild air puff to the neck of the mouse (0.7 bar) and access to water was denied. Two cages were run in parallel: one with two male *Otof*-KO siblings, and one with five *Otof*-KO females (age 14.4 ± 2.4 weeks), all with a successful ABR rescue (click threshold 50 dB). Sex matched WT controls (C57Bl/6 background, 16.0 ± 2.6 weeks) were measured as a separate dataset.

### Data Analysis and Figure Preparation

**Figure 1:**
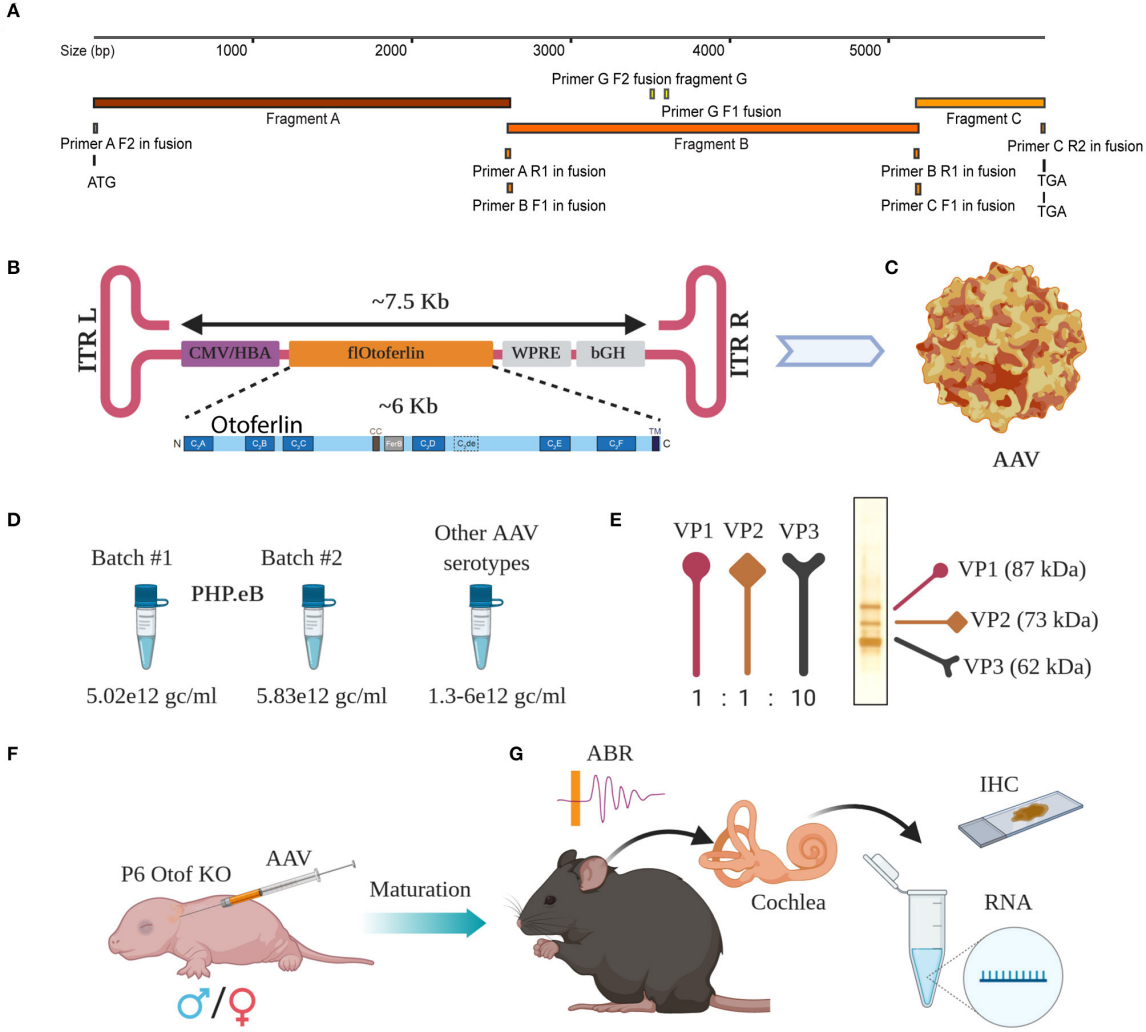
Overload AAV construct design and AAV preparation. **(A)** Cloning strategy with primers located at their bindings sites. **(B)** Schematic representation of *fl-Otof*-CDS-overload construct (inset: domain overview) flanked by two inverted terminal repeats (ITRs). **(C)** Surface model of a single AAV particle. **(D)** Comparable titers from several independent AAV preps demonstrate batch to batch consistency. **(E)** Viral capsid proteins VP1, VP2, and VP3 with their expected ration 1:1:10 in one representative virus preparation after conventional silver staining. **(F)** Purified AAV particles carrying *fl-Otof*-CDS were used in postnatal injections of P6 mice *via* the round window membrane. **(G)** Once matured, injected animals were further studied in various experimental settings including ABR recordings, behavioral tests, immunohistochemistry, and RNA isolation for sequencing purposes.

**Figure 2:**
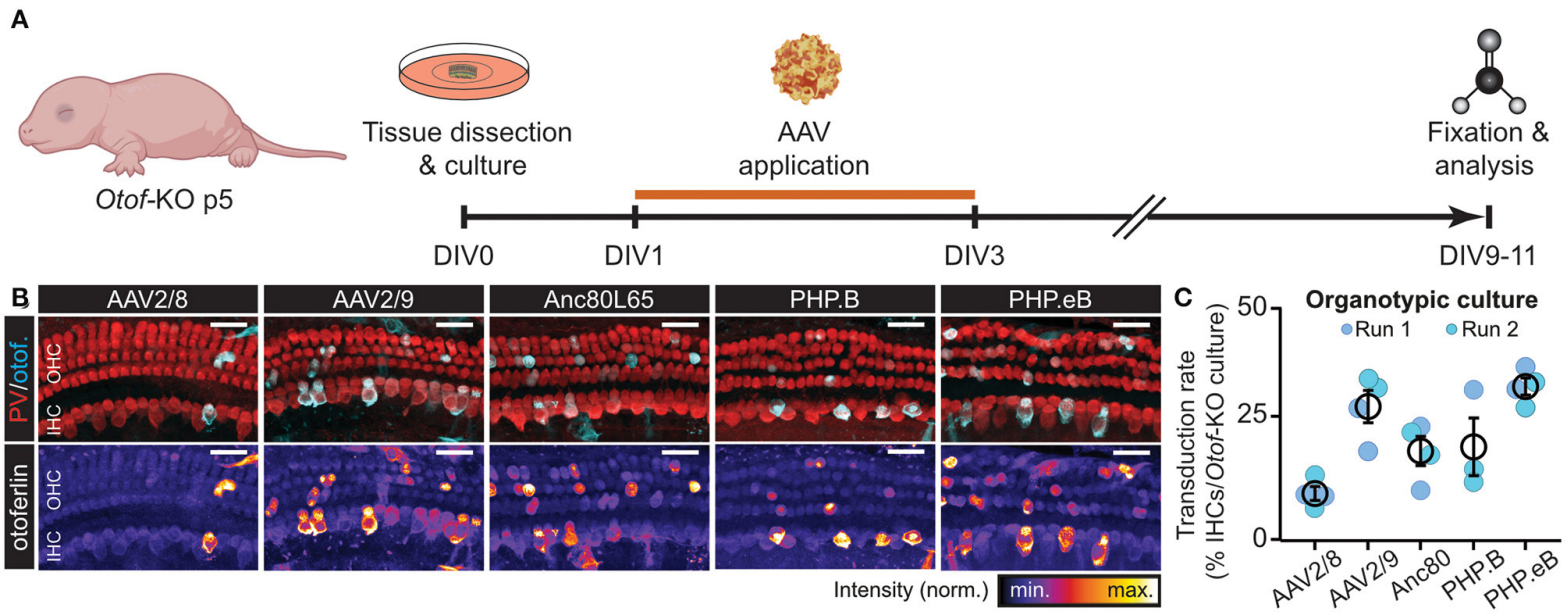
*fl-Otof-*overloaded AAVs can reproducibly transduce hair cells *in vitro*. **(A)** Schematic illustration of the experimental paradigm: cochlear explant cultures were dissected from p5 *Otof-*KO mice and kept in culture for up to 11 days. On the first day *in vitro* (DIV) a range of different *fl-Otof*-AAV variants were applied for 48 h in cell culture medium. After 6–8 days, the explant cultures were fixed and immunostained for the hair cell-specific cytosolic Ca^2+^ buffer parvalbumin (PV) and otoferlin. **(B)** Representative maximum projections of cochlear explant cultures after transduction with the indicated AAVs. The respective otoferlin immunofluorescence is presented with an intensity-coded look-up table below the merged images for clarity. **(C)** Quantification of the respective transduction rates for each tested AAV capsid carrying *fl-Otof-*CDS (3–4 explants from 3 to 4 animals per condition from two independent culture runs). Scale bars in **(B)**: 25 μm.

Data was analyzed and figures were prepared using Microsoft Excel, Igor Pro and custom written Matlab routines. Figures were assembled using Adobe Illustrator, Affinity Designer, SnapGene and BioRender.com software (Figures 1, 2, **5** and Supplementary Figure 3 were partially Created with BioRender.com).

## Results

### AAVs Can Efficiently be Overloaded With the Full-Length *Otof* Coding Sequence

Successful packaging of fragments larger than 4.7 kb into standard AAVs had been reported in the past (Allocca et al., 2008; Grose et al., 2012; Hirsch et al., 2013; Pryadkina et al., 2015). Encouraged by these results, we packaged the *fl-Otof*-CDS and regulatory elements into various AAVs. When designing the experimental strategy, we decided against attaching an additional fluorescent reporter tag considering the large size of the *fl-Otof*-CDS. Therefore, analysis of transduction efficiency relied on post-mortem otoferlin immunohistochemistry. The *fl-Otof*-CDS used for this study was generated in several steps by performing in-fusion cloning strategies (Figure 1A, for details see Material and Methods section and Appendix A1). Integrity of the obtained construct was validated by Sanger sequencing. In the final construct, we decided to employ a hybrid CMV/hbA promoter that is known to drive strong transgene expression in mammalian tissues (Figure 1B). Although this promoter does not offer IHC-specificity *per se*, we expected that the combination of capsid, promoter and target gene would favor expression in IHCs (Akil et al., 2012; Jung et al., 2015; Al-Moyed et al., 2019). Moreover, due to otoferlin's highly specific function in the final steps of IHC exocytosis, we reasoned that potential ectopic expression in adjacent supporting cells, etc. should not interfere with hearing restoration in *Otof-*KOs. In addition, for enhancement of expression the Woodchuck Hepatitis Virus Posttranslational Regulatory Element (WPRE) and for stability of the transcript the bovine growth hormone (bGH) polyadenylation sequence were included between the inverted terminal repeats (ITRs). The entire sequence between the two ITRs was around 7.5 kb (Figure 1B and Supplementary Figure 1B) exceeding the regular packaging capacity of AAVs (4.7 kb). We successfully packaged the construct into the novel virus serotype PHP.eB with consistent titers (Figures 1C,D and Supplementary Table 1). Packaging was also successful in our hands with several other AAV serotypes with reproducible titers (Figure 1D). We checked all virus preparations for the purity by conventional silver staining and, in most cases, detected excellent purity with three most important viral capsid proteins VP1, VP2, and VP3 (Figure 1E, one representative virus preparation). The virus preparations were then employed for transduction efficiency assessment on otoferlin-deficient hair cells—initially in organotypically-cultured organs of Corti and subsequently in cochleae of newborn *Otof-*KO mice (Figure 1F). Animals typically recovered well following injection and assessment of hearing as well as expression analysis were performed 4–5 weeks and 5 months later (Figure 1G).

### *fl-Otof*-Overloaded AAVs Transduce Hair Cells *in vitro*

To test if the *fl-Otof-*CDS-overloaded AAVs are capable of transducing mammalian IHCs, we first tested our different virus preparations on organotypically-cultured cochlear explants of *Otof-*KO mice (Figure 2). In these experiments, we dissected the organs of Corti at postnatal day (p) 5, cultured them overnight in serum-free medium and subsequently incubated them for 48 h with AAV-containing growth medium. After additional 6–8 days *in vitro* (DIV), we fixed the explants and processed them for immunohistochemistry, finally probing them with an otoferlin-specific antibody (Figures 2A,B). Here, all AAV preparations successfully and reproducibly transduced cultured IHCs—though at varying degrees—as well as some OHCs and various types of supporting cells, with AAV2/8 consistently performing the worst and PHP.eB the best (Figure 2C; mean transduction rates: AAV2/8: 7.1 ± 1.6% [*n* = 4 organs from *N* = 4 animals]; AAV2/9: 27.3 ± 3.7% [*n* = 4 organs from *N* = 4 animals]; Anc80L65: 17.0 ± 3.6% [*n* = 4 organs from *N* = 4 animals]; PHP.B: 17.9 ± 6.7% [*n* = 3 organs from *N* = 3 animals] and PHP.eB: 31.9 ± 2.0% [*n* = 4 organs from *N* = 4 animals]). This finding shows that the principle of AAV-overloading is not limited to a single serotype/capsid variant and reproducible across trials.

### *fl-Otof*-Overloaded AAVs Successfully Transduce IHCs of Neonatal Mice *in vivo*

**Figure 3:**
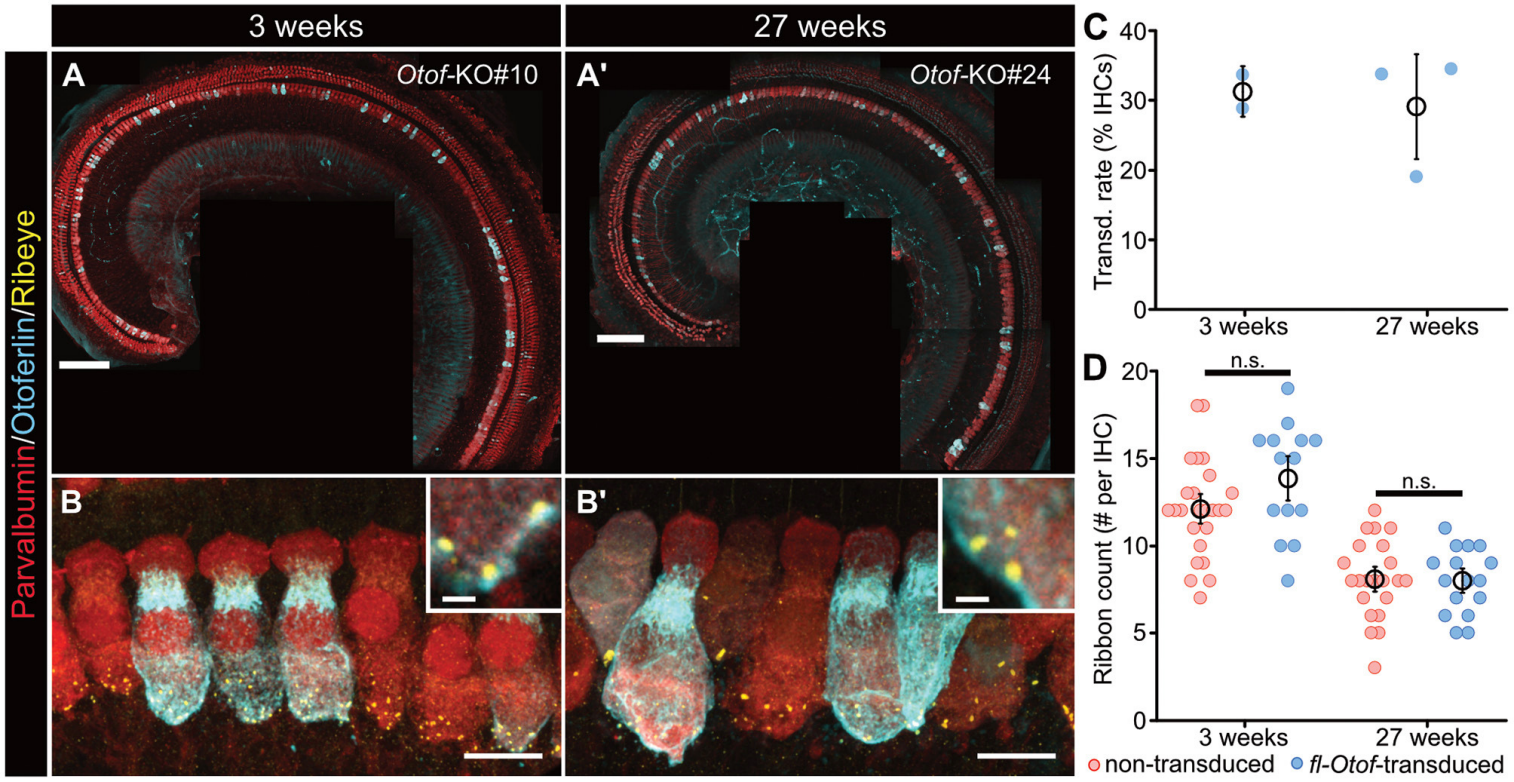
*In vivo* transduction efficiency and synapse counts of *fl-Otof-*transduced IHCs. **(A,A')** Representative confocal maximum projections of acutely-dissected apical turn organs of Corti from **(A)** a p16 and **(A')** a 27 weeks-old *Otof-*KO mouse after PHP.eB-injection at p5 to assess viral transduction rates. Hair cells were immunostained for the cytosolic Ca^2+^-buffer parvalbumin and counterstained against otoferlin. **(B,B')** Higher magnification confocal maximum projections of IHCs immunohistochemically-labeled against parvalbumin, otoferlin, and ribeye to establish ribbon counts per IHC in **(B)** the young and **(B')** older age group. **(C)** Quantification of IHC transduction rates: mean ± SEM and estimate from individual mice (blue spheres). **(D)** Ribbon counts in *fl-Otof*-transduced and non-transduced IHCs. No statistically significant differences could be detected between *fl-Otof-*transduced and non-transduced IHCs in the different age groups (Shapiro-Wilk normality test; one-way ANOVA with *post-hoc* Tukey). Scale bars in **(A,A')**: 100 μm, in **(B,B')**: 10 μm; insets: 1 μm.

Encouraged by these promising *in vitro* results, we now selected the best-performing PHP.eB virus construct for subsequent *in vivo* analyses. Initially, we evaluated the *in vivo* transduction efficiency of the rAAV PHP.eB vectors carrying *fl-Otof via* immunohistochemical analysis (Figure 3). Importantly, cochlear and vestibular anatomy appeared to remain largely intact in the injected ears, with no obvious signs of persistent inflammation, malformation or other morphological anomalies, suggesting that neither the surgical procedure nor AAV transduction itself did exert any major adverse effects. In an attempt to address stability of transgenic otoferlin expression, we dissected and analyzed the apical turns of two distinct age groups (Group 1: directly post-hearing onset, i.e., 3 weeks; Group 2 at ~6 months old; Figures 3A,A') and found a comparable transduction rate in both age groups of ~30% (Group 1: 31.3 ± 2.4%, *N* = 2; Group 2: 29.1 ± 5.0%, *N* = 3; Figure 3C). Interestingly, our data indicate that insert size is indeed a main determinant of transduction rate and/or transgenic protein expression, since a truncated and GFP-tagged, C-terminal fragment of otoferlin (“*miniOtof* ”) that was packaged using the identical capsid/promoter construct as the *fl-Otof-CDS*—and thus solely differs in insert size—achieved much higher transduction rates. Yet, consistent with a previous report using a similar approach (Tertrais et al., 2019) our *mini-Otof* construct failed to restore hearing in *Otof-*KO mice (Supplementary Figure 3).

Since it is well-established that afferent synapses of *Otof-*KOs degenerate with advancing age (Roux et al., 2006), we also assessed synapse numbers in *fl-Otof-*transduced and adjacent non-transduced IHCs using immunohistochemical analysis (Figures 3B,B'). Consistent with previous studies that also aimed to gene-therapeutically restore hearing in *Otof-KOs via* early postnatal injections *via* a dual-AAV approach (Akil et al., 2019; Al-Moyed et al., 2019), our analysis showed a clear decrease of ribbon numbers in aging *Otof-*KO IHCs that was comparable between transduced and non-transduced IHCs and, hence, could not be recovered with PHP.eB-*fl-Otof* delivery at p5-7 (Figure 3D; non-transduced 3 weeks-old 12.1 ± 0.6 synapses/IHC, *n* = 25, *N* = 2; *fl-Otof*-transduced: 13.9 ± 0.9 synapses/IHC, *n* = 14, *N* = 2; non-transduced 27 weeks-old 8.1 ± 0.5 synapses/IHC, *n* = 22, *N* = 2; *fl-Otof*-transduced: 8.0 ± 0.5 synapses/IHC, *n* = 16, *N* = 2; one-way ANOVA with *post-hoc* Tukey).

**Figure 4:**
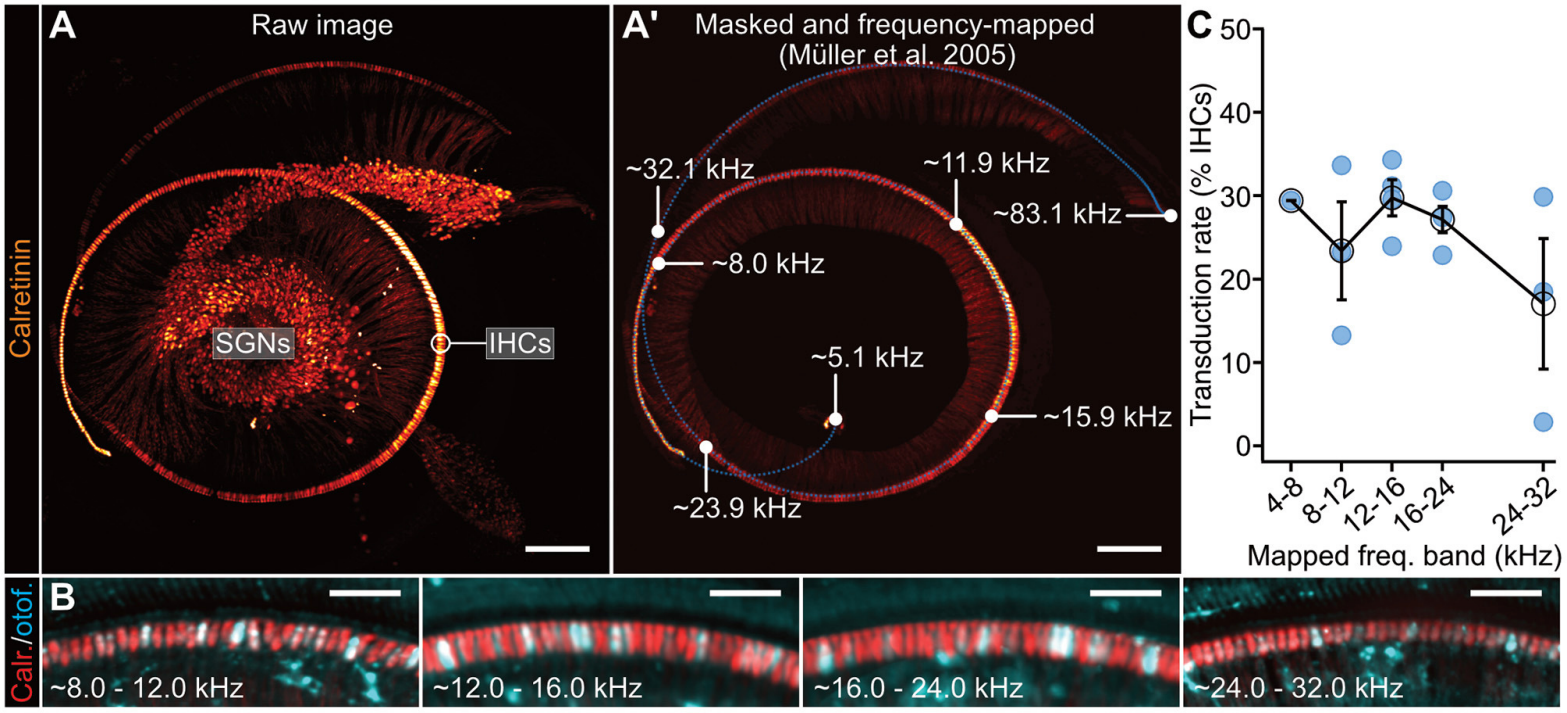
Tonotopic analysis of *fl-Otof* transduction rates. **(A)** Representative 3D maximum projection of a lightsheet fluorescence microscopy data set of an *Otof-*KO mouse cochlea (p24, injected with PHP.eB-CMV/HBA-fl-Otof at p6). For context marking, IHCs and SGNs—including the afferent auditory nerve fibers—were stained against the cytosolic calcium buffer calretinin. **(A')** Tonotopic mapping of the masked IHC row based on Greenwood's function (Greenwood, 1961). Parameters were set according to Müller et al. (2005) resulting in a frequency range from a minimum of 5.1 kHz to a maximum of 83.1 kHz. The blue dotted line indicates reference points for the frequency-mapping algorithm in which a total of 500 coordinates were interpolated to fit the cochlear spiral. Selected frequency labels indicating the projected boundaries of the defined frequency bands (white dots) are indicated in the figure panel. Please note that the 4–8 kHz region was structurally less well-preserved in several of the processed cochleae, thus preventing meaningful analysis of this particular frequency band using this approach. **(B)** Representative maximum projections of the indicated frequency regions at higher magnification. IHCs were immunohistochemically-labeled against calretinin and otoferlin to assess viral transduction rates. **(C)** Quantification of IHC transduction rates in the indicated frequency regions using this approach: mean ± SEM and estimate from individual mice (blue spheres). Scale bars. **(A)** 200 μm; **(B)** 50 μm.

To now further extend this analysis and establish if *fl-Otof* transduction rates varied along the tonotopic axis, we set out to determine *fl-Otof* transduction rates across the entire cochlear spiral, using chemical tissue clearing and subsequent lightsheet imaging approach of the whole immunostained cochlea *in situ* (Figure 4). In these experiments, AAV-injected cochleae were harvested after 3–4 weeks post injection and processed for immunohistochemistry. We labeled IHCs against otoferlin and the cytosolic Ca^2+^-buffer calretinin as a context marker (Figure 4A). Calretinin immunoreactivity was then used to define our region of interest for analysis and served to determine reference points for frequency-mapping according to a previously published model [Figure 4A'; (Müller et al., 2005)]. Subsequently, we counted otoferlin-positive IHCs within the calculated frequency bands (Figures 4B,C). Consistent with our confocal analysis of the apical cochlear turn, these data suggest a remarkably stable transduction rate of ~23–30% until the 24 kHz region (~4–8 kHz: 29.4%, limited to *N* = 1 due to limited structural preservation; ~8–12 kHz: 23.4%, *n* = 3 cochleae; ~12–16 kHz: 29.7%, *n* = 4 cochleae; ~16–24 kHz: 27.1%, *n* = 4 cochleae). At higher frequencies beyond ~24 kHz, the transduction rate appeared to drop below 20% (~24–32 kHz: 17.1%, *n* = 3 cochleae). If this latter observation is indeed of biological significance or rather a technical artifact due to poorer antibody penetration, etc. remains to be determined.

**Figure 5:**
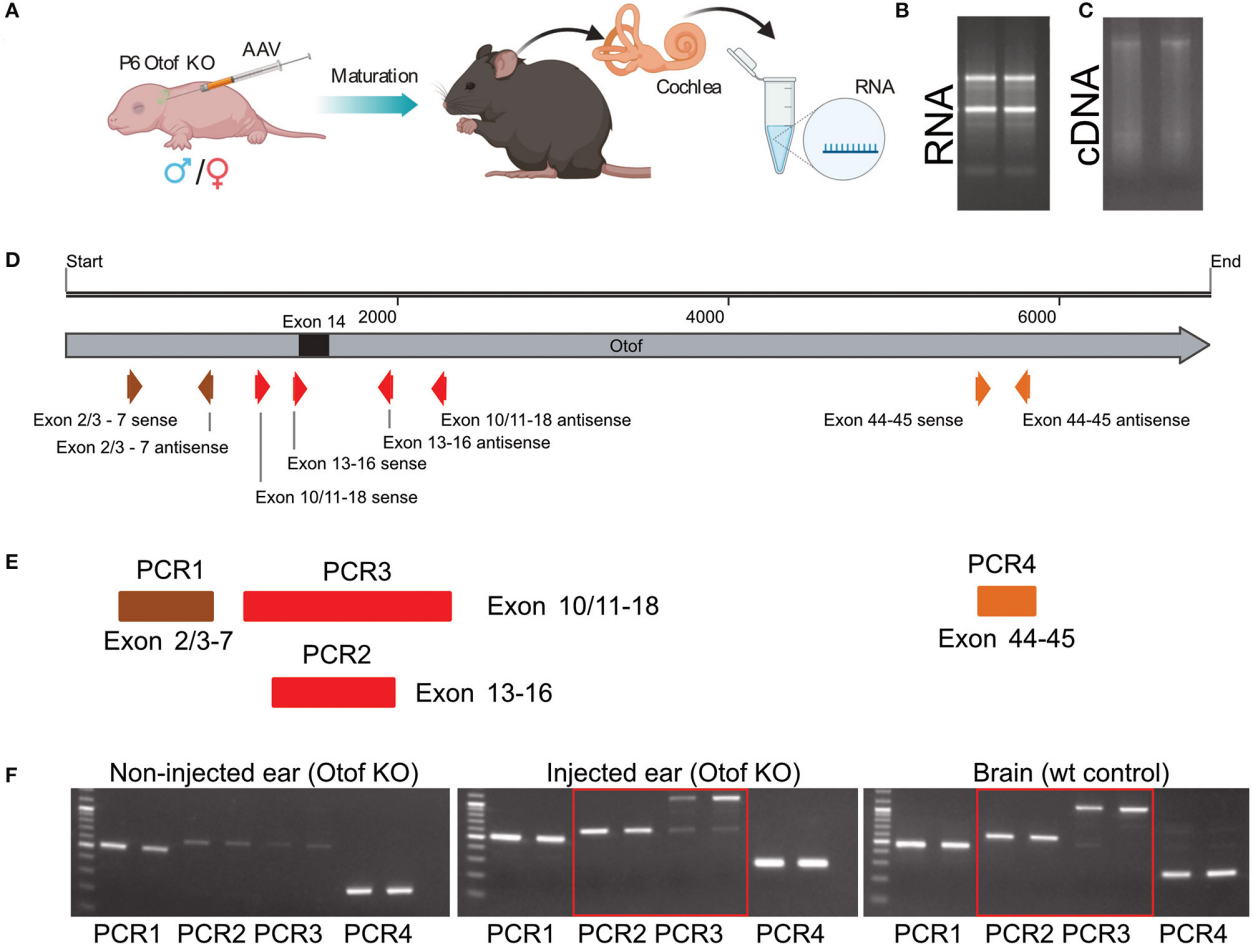
cDNA sequencing with primers specific to various distinct otoferlin sequences. **(A)** Schematic overview of the injection procedure and subsequent experimental procedure. **(B)** RNA isolation from cochleae. **(C)** cDNA reverse transcription of RNA. **(D)** Full-length otoferlin sequence with all exons and primer binding sites used for PCRs. Exon 14, which was removed in *Otof*-KO mice is labeled as a black square. **(E)** PCR runs covering various otoferlin exons. **(F)** Obtained results from non-injected as well as injected ears and wild-type mice brain using the primers specific for various otoferlin sequences.

Next, total RNA isolation from *fl-Otof*-injected and contralateral non-injected cochleae (*n* = 5, Figure 5) confirmed full-length wild-type otoferlin expression in the injected cochleae of these *Otof*-KO mice, as we were able to detect several otoferlin regions covering multiple exons from the N- to C-terminus using PCRs with region-specific primers (Appendix A2). As the KO-strategy deletes exon 14 (Figure 5D, black square), the finding of residual otoferlin mRNA fragments from PCR1 and PCR4 in the non-injected ear is expected, while the faint bands of PCR2 and 3 likely relates to spread of virus during the early postnatal injections.

### *fl-Otof*-Overloaded AAVs Partially Restore Hearing in *Otof*-KO Mice

**Figure 6:**
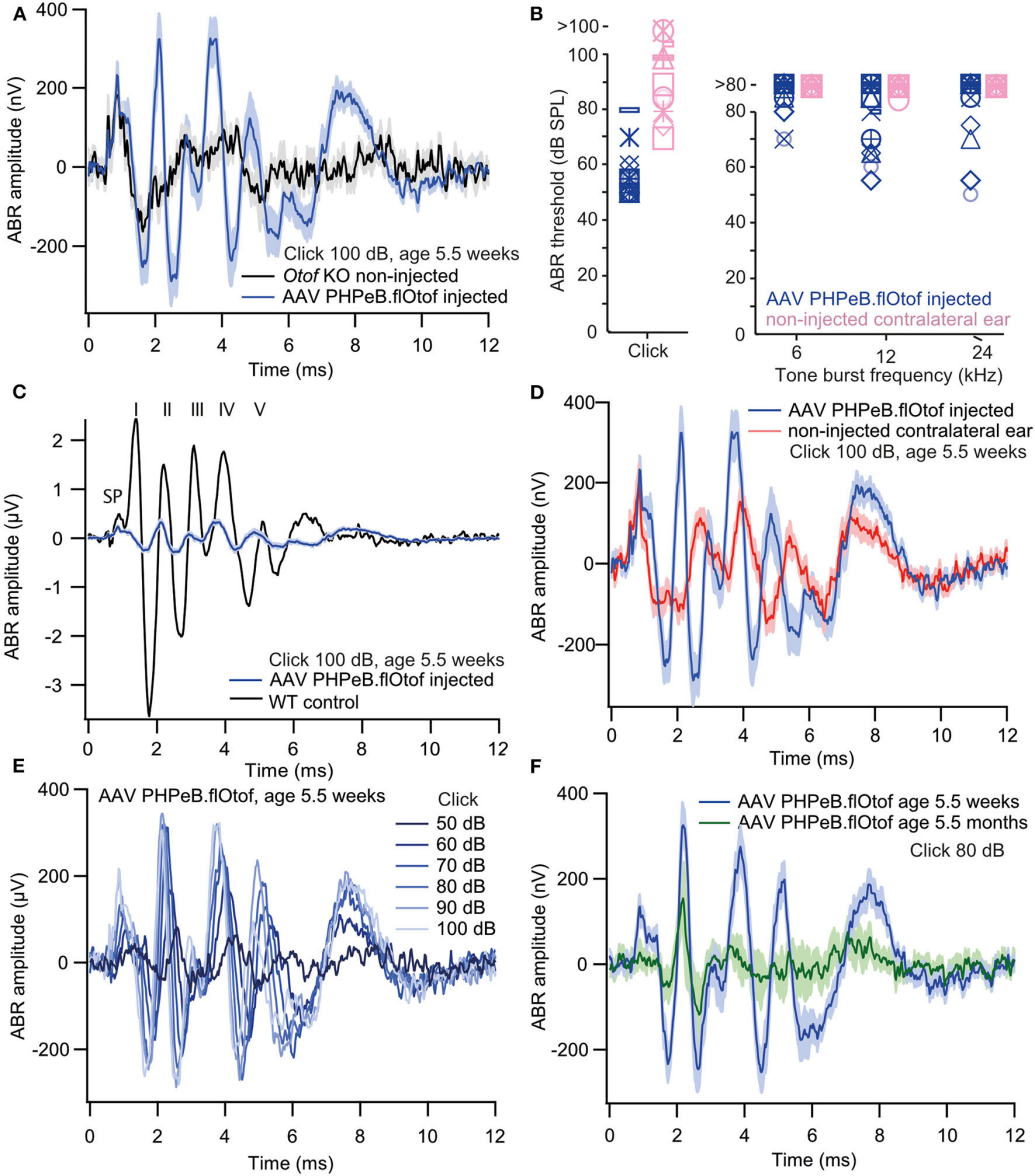
Rescue of hearing function in *Otof*-KO mice: auditory brainstem responses. **(A)** Grand averages of ABR waveforms 100 dB Click stimulation in *Otof*-KO mice following injection of PHP.eB *fl-Otof* AAV in one ear (blue, *n* = 18, excluding the seven animals with no reproducible click response) and non-injected *Otof*-Ko mice (black, *n* = 8). **(B)** ABR thresholds to clicks and 6, 12, and 24 kHz tone bursts in injected and contralateral ears (*n* = 18). Each symbol represents one animal. **(C)** The amplitude of rescued ABR waveforms [blue, same as in **(A)**] is much smaller than in a representative trace from a single non-injected C57Bl/6 WT mouse (black) but latencies are comparable. SP denotes the summating potential, the presumptive inner hair cell receptor potential. Roman numerals on top indicate ABR waves I-V (Jewett et al., 1970). **(D)** ABRs from successfully rescued [blue, same as in **(A,C)**] and non-injected contralateral ears of the same animals (red) and a are shown for comparison. **(E)** Amplitude growth function of rescued ears (grand averages of ABR waveforms to 50–100 dB click stimulation, *n* = 17). **(F)** Grand averages of ABR waveforms to 80 dB click stimulation from ears with successful viral rescue, measured at an age of 5.5 weeks (blue, *n* = 18) and 5.5 months (green, *n* = 8). All blue traces are from the same dataset of PHP.eB *fl-Otof* AAV injected ears, age 5.5 weeks. Shaded areas represent means ± SEM.

Next, we injected *Otof-*KO mice with *fl-Otof*-PHP.eB at p5-7 and then measured the auditory brainstem response (ABR) at the age of 5.5 ± 0.2 weeks. In these experiments, we found partly recovered ABRs to click stimulation in 18/25 tested mice with an average threshold of 58 ± 3 dB. In response to tone burst stimulation, only 10 injected ears showed reproducible responses (Figures 6A,B). As expected for a partial rescue, the ABR wave amplitudes were smaller than in WT mice. Like in other mutant mice with deficits of the IHC ribbon synapse (Buran et al., 2010; Strenzke et al., 2016), waves II, IV, and V were preserved better than waves I and III (Figure 6C). Non-injected contralateral ears also showed reproducible responses to click stimulation with an average threshold of 93 ± 3 dB (Figure 6D). We believe that this response mainly reflects viral spread to the non-injected ear during early postnatal development but cannot completely exclude acoustic cross-talk. The amplitude of click-evoked ABRs rose with intensity (Figure 6E) and approached its maximum from 74 ± 2 dB (*n* = 14). This suggests that like in WT, the recruitment of IHC/SGNs and the activation of individual SGNs saturates at high intensities. Partial viral rescue of hearing function persisted for several months, as demonstrated by sizeable ABRs recorded at an age of 5.5 ± 0.2 months (Figure 6F). Other overloaded AAVs also partially rescued hearing function, albeit with a lower success rate (Supplementary Table 1 and Supplementary Figure 4).

**Figure 7:**
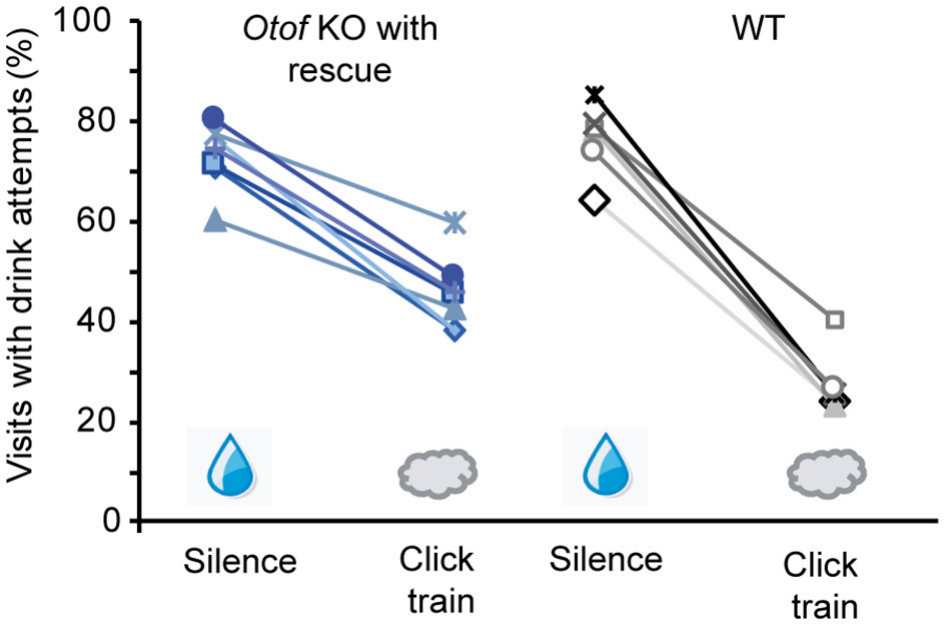
Rescue of hearing function in *Otof*-KO mice: operant conditioning. Successful discrimination between silence (water access) and 100 dB click trains (no water access, air puff punishment) was achieved in all six tested *Otof*-KO mice with successful viral rescue (blue colors), albeit with lower discrimination performance than in five C57Bl/6 WT controls (black and gray colors).

Finally, to assess the restoration of auditory perception in PHP.eB *fl-Otof* AAV-treated mice, we used operant conditioning under automated control in the “intellicage” system (de Hoz and Nelken, 2014; Strenzke et al., 2016) and trained mice to discriminate between click trains and silence (Figure 7). During 10% of visits to the experimental “corner,” 100 dB click trains were presented and drink attempts were punished by an air puff. In 90% of visits, no sound was presented and access to water was allowed. All six tested *Otof*-KO mice with partial rescue of cochlear function (ABR thresholds of 50 dB) successfully learned the task with an average discrimination performance of 27.6 ± 3.0%. Matched normal-hearing C57Bl/6 WT control mice had a slightly better discrimination performance of 49.1 ± 3.5%.

## Discussion

Here, we investigated the potential of single AAV overloading for the future gene therapy of otoferlin-related auditory synaptopathy. Packaging of *fl-Otof-*CDS into several AAV variants was feasible at high titers (10^12^ genome copies/ml) and purities. While all tested overloaded AAV variants successfully transduced IHCs *in vitro* and led to otoferlin expression in IHCs of cultured cochlear explants, the synthetic PHP.eB *fl-Otof* AAV performed the best in our initial screen. Subsequent *in vivo* analyses revealed that (i) the efficiency of otoferlin expression, (ii) specificity of transgene expression in IHCs, and (iii) rescue of auditory brainstem responses in *Otof*-KO mice upon early postnatal injection of PHP.eB *fl-Otof* AAV appeared largely comparable to that previously reported for the dual-AAV approach using the same age of injection (Al-Moyed et al., 2019), rendering our simplified approach highly interesting for further clinical development. Finally, for the first time to our knowledge, using operant conditioning, we could further demonstrate auditory percepts in *Otof*-KO mice that had undergone viral gene therapy.

The small size and consequently good tissue penetration, low immunogenicity, lack of pathogenicity and episomal (extrachromosomal) DNA organization in the host cell make AAVs attractive vectors for gene therapy of sensory disorders (recently reviewed in Kleinlogel et al., 2020). For example, the recent approval of AAV-RPE65 gene therapy (Luxturna) of Lebers' congenital amaurosis 2, AAV-mediated vision restoration has been hailed a major breakthrough in this field (Bennett, 2017; Ledford, 2017).

In the past, coding sequences exceeding the transgene capacity of AAVs (4.7 kb) have been placed into lentiviruses or adenoviruses, which are plagued by issues such as insertional mutagenesis, mobilization, and vector replication or immunogenicity (Kleinlogel et al., 2020). More recently, split-AAVs (Duan et al., 1998, Duan et al., 2001; Maddalena et al., 2018) and “overloaded AAVs” (Allocca et al., 2008; Grose et al., 2012; Hirsch et al., 2013; Pryadkina et al., 2015) have been employed in preclinical studies to circumvent these issues. In split-AAVs, the expression cassette is separated into two (dual-AAV) or three (triple-AAV) pieces, containing highly recombinogenic sequences and then packaged into two or three AAVs, respectively. Upon co-transduction the contained DNAs recombine into one single CDS. Dual AAV vectors have been successfully employed for expressing full-length otoferlin in IHCs of *Otof-*KO mice (Akil et al., 2012; Al-Moyed et al., 2019) achieving expression in up to 50% [injection at p6-7, (Al-Moyed et al., 2019)] and above 80% [injection at p17 or p30, (Akil et al., 2019)] of IHCs. Although our AAV overloading approach appears to be inferior in regards to transduction efficiency at this stage, it is highly encouraging that we observed homogeneous transduction rates along the tonotopic axis—at least until the 24 kHz range. Here, the large insert size seems to be the main limiting factor of transgenic otoferlin expression, as a substantially smaller mini-Otoferlin construct—packaged into the exact same promoter/capsid combination—achieved drastically improved AAV transduction rates. Here, we used the efficient CMV-hbA promoter which despite its broad scope for driving expression in various cells, led to relatively specific transgenic expression of otoferlin in IHCs. In future studies, higher viral titers, IHC-specific promoters—such as Myosin 15—or combinatorial virus application approaches (Lee et al., 2020) may help improve the efficiency and specificity of transgenic expression of otoferlin in IHCs. In addition, some other approaches coming into the focus of gene therapy studies—such as non-integrating lentiviruses (Suwanmanee et al., 2014; Snowball et al., 2019) or novel oversized AAVs, namely XL-AAVs (Ding and Gradinaru, 2020), with much larger packaging capacity—might overcome this limitation and finally lead to the full functional rescue of otoferlin-related hearing deficits.

The need to combine multiple distinct rAAVs presents an additional biosafety burden that may hinder clinical translation of such a treatment. This issue motivated us to explore the potential use of “overloaded AAVs” for future gene therapy of otoferlin-related auditory synaptopathy. Here, the omission of a fluorescent marker from the transgene is beneficial from a translational point of view, but presented a drawback for this preclinical study as it hampered the easy identification and more detailed analysis of IHC exocytosis by cell physiology. This was particularly critical given that otoferlin expression was *post-hoc* detectable in, on average, only a third of the IHCs with the present protocol. Future studies should compare the efficiency of both, AAV-overloading and split-AAV approaches using the same serotype/capsid and promoter combination as well as equal titers and virus administration protocols. Cell-physiological analysis, an important objective for future work, will be facilitated by employing a small tag for easy identification of transduced IHCs. Moreover, next to assessing otoferlin protein expression at various time points, single IHC sequencing should ideally be used to reveal the *Otof* DNAs generated by both approaches.

Finally, the extent of hearing restoration should be scrutinized using several complementary approaches. Partial restoration of synaptic function achieved with AAV-mediated *Otof* gene transfer into IHCs of *Otof-*KO mice has been demonstrated by recordings of ABRs (Akil et al., 2012; Al-Moyed et al., 2019 and this study), patch-clamp recordings from IHCs (Al-Moyed et al., 2019) and, finally, behavioral experiments (this study). Clearly, these results are very promising and indicate that clinical translation might eventually enable unaided hearing in people affected by *OTOF* mutations. Given the auditory synaptopathy phenotype (Moser and Starr, 2016) that is sensitively reported by lack or impairment of the population responses of SGNs [ABRs and electrocochleography (Moser and Starr, 2016)], the remaining ABR deficit might lead to an overestimation of the hearing impairment. Indeed, normal acoustic sensitivity has been reported for patients with some *OTOF* missense mutations (for review see: Santarelli et al., 2015) and the respective mouse model (Strenzke et al., 2016). Obviously, the more functional otoferlin remains present in case of missense mutations affecting the abundance of otoferlin at the plasma membrane—such as those causing the Ile515Thr substitution (Varga et al., 2006) or the Ile1967 deletion (Vogl et al., 2016)—the more likely it is that the transgenic supplementation of otoferlin expression in IHCs is going to restore normal hearing. Future preclinical work should aim to increase the certainty of predicting the outcome of hearing restoration in the clinical application of gene-therapeutic approaches to otoferlin-related auditory synaptopathy. For example, studies of temporal processing and of loudness adaptation will be helpful to assess potential remaining deficits (Strenzke et al., 2016). Eventually, work with species more closely related to humans, such as non-human primates, will help to prepare and accompany clinical trials.

## Data Availability Statement

The raw data supporting the conclusions of this article will be made available by the authors, without undue reservation.

## Ethics Statement

The animal study was reviewed and approved by board for animal welfare of the University Medical Center Goettingen and the animal welfare office of the state of Lower Saxony (Animal protocol Nr. 14_1391).

## Author Contributions

VR, CV, NS, and TM designed the study and wrote the manuscript with contributions of all other authors. VR, CV, IB, ND, CD-A, AT, TW, and KK performed experiments and/or analyzed data. All authors contributed to the article and approved the submitted version.

## Conflict of Interest

The authors declare that the research was conducted in the absence of any commercial or financial relationships that could be construed as a potential conflict of interest.
