# Supplementary material for: Overloaded Adeno-Associated Virus as a Novel Gene Therapeutic Tool for Otoferlin-Related Deafness
Source: Front Mol Neurosci. 2021 Jan 7;13:600051. doi: 10.3389/fnmol.2020.600051 (PMC7817888; doi:10.3389/fnmol.2020.600051)
Supplement: Supplementary file 1 [file Table_1.DOCX]

Overloaded adeno-associated virus as a novel gene therapeutic tool for otoferlin-related deafness

Vladan Rankovic^1,2,#,*^, Christian Vogl^1,3,4,#,*^, Nele M. Dörje^1,3^, Iman Bahader^4,5^, Carlos J. Duque-Afonso^1,6,7^, Anupriya Thirumalai^1^, Thomas Weber^1^, Kathrin Kusch^1^, Nicola Strenzke^4,5^, and Tobias Moser^1,4,6,7,8,9*^

**Appendix:**

**A1.** Target vector, fragments & cloning primers with overlaps for in-fusion cloning.

**Fragment A**

A_F2: AATTCAAGCTGCTAGC***ATG***GCCCTGATTGTTCACC

A_R1: CGTTTGTTGTTGCTCATCATCCAAATG

**Fragment B**

B_F1 GAGCAACAACAAACGTATCGCCTATGC

B_R1 GCAGCTCGTACTTCTTGGGTTTCCTG

**Fragment C**

C_F1 AGAAGTACGAGCTGCGGGTCATCGTG

C_R2 GATTATCGATAAGCTT***TTA***GGCCCCTAGGAGCTT

**Fragment G**

G_F1 CGGACTCAGATCTCGCTGGTCAAGTGGTTTGAAGTG

B_R1 GCAGCTCGTACTTCTTGGGTTTCCTG

**Fragment C2**

C_F1 AGAAGTACGAGCTGCGGGTCATCGTG

C_R1 AGAATTCGAAGCTTG***TTA***GGCCCCTAGGAGCTT

Table 1. Cloning primers

(5’-3’ orientation, overlaps underlined, start and stop codon for transcription bold/italics)

**A1.1.** In-Fusion cloning strategy with fragment overlaps.

EcoRI NheI HindIII

5’…GCGGCCGCCCAAAGAATTCAAGCTG’ AGCTTATCGATAATCAAC… 3’

3’…CGCCGGCGGGTTTCTTAAGTTCGACGATC 5’ ATAGCTATTAGTTG… 5’

(region of interest for in fusion cloning)

Linearized Target vector pAAV (NheI/HindIII)

-Forward primer A_F2 and overlap:

X addition restores NheI site

Overlap-> 5’ AATTCAAGCTGCTAGC 3’

EcoRI NheI HindIII

5’…GCGGCCGCCCAAA**GAATT**CAAGCTG’ AGCTTATCGATAATCAAC… 3’

3’…CGCCGGCGGGTTTCTTAAGTTCGACGATC 5’ ATAGCTATTAGTTG… 5’

A_F2: AATTCAAGCTGCTAGC**ATG**GCCCTGATTGTTCACC

-Reverse primer C_R2 and overlap:

EcoRI NheI HindIII

5’…GCGGCCGCCCAAA**GAATT**CAAGCTG’ AGCTTATCGATAATCAAC… 3’

3’…CGCCGGCGGGTTTCTTAAGTTCGACGATC 5’ ATAGCTATTAGTTG… 5’

3’ TTCGAATAGCTATTAG 5’ <- overlap

X addition restores HindIII site

C_R2 GATTATCGATAAGCTT**TTA**GGCCCCTAGGAGCTT

**A1.2.** Primers with split overlaps.

**Overlap arms of 15bps were split between Primers at the overlaps between**

**adjacent fragments A and B and B and C respectively**

**Fragment A 3’end Fragment B 5’ end**

8bp 7bp

5’ …GTGTTCATTTGGATGATGAGCAACA ACAAACGTATCGCCTATGCCCGCG…

3’ …CACAAGTAAACCTACTACTCGTTGT TGTTTGCATAGCGGATACGGGCGC…

8bp

-Forward primer B_F1: GAGCAACAACAAACGTATCGCCTATGC

7bp

-Reverse primer A_R1: CGTTTGTTGTTGCTCATCATCCAAATG

**Fragment B 3’ end Fragment C 5’ end**

8bp 7bp

5’ …CCCAGGAAACCCAAGAAGTAC GAGCTGCGGGTCATCGTGTGGAACAC…

3’ …GGGTCCTTTGGGTTCTTCATG CTCGACGCCCAGTAGCACACCTTGTG…

8bp

-Forward primer C_F1: AGAAGTACGAGCTGCGGGTCATCGTG

7bp

-Reverse primer B_R1: GCAGCTCGTACTTCTTGGGTTTCCTG

**A2.** Primers used for cDNA sequencing.

**PCR1**

Otoferlin_cDNA_Exon2/3_to_Exon7

PS_38016_26799_**506bp**_a 55% GC

PS_38016_26799_**506bp**_b 55% GC

**PCR2**

Otoferlin_cDNA_Exon13_to_Exon16

PS_29627_37305_**587bp**_a 55% GC, or delta 384bp (Ex14Ex15-transv1) = **203bp**

PS_29627_37305_**587bp**_b 55% GC

**PCR3**

Otoferlin_cDNA_Exon10/11_to_Exon18

PS_13685_34228_**1114bp**_a 55% GC, or delta 384bp (Ex14Ex15-transv1) = **730bp**

PS_13685_34228_**1114bp**_b 55% GC

**PCR4**

Otoferlin_cDNA_Exon44_to_Exon45

PS_37087-2_35765_**264bp**_a 57% GC

PS_37087-2_35765_**264bp**_b 57% GC

**A3.** CLUSTAL O(1.2.4) multiple sequence alignment. Protein alignment between mouse and human Otoferlin sequences (NCBI accession numbers mouse NP_001093865.1 (top) and human NP_001274418.1 (bottom).

Mouse_var1 MALIVHLKTVSELRGKGDRIAKVTFRGQSFYSRVLENCEGVADFDETFRWPVASSIDRNE 60

Human_var5 MALLIHLKTVSELRGRGDRIAKVTFRGQSFYSRVLENCEDVADFDETFRWPVASSIDRNE 60

***::**********:***********************.********************

Mouse_var1 VLEIQIFNYSKVFSNKLIGTFCMVLQKVVEENRVEVTDTLMDDSNAIIKTSLSMEVRYQA 120

Human_var5 MLEIQVFNYSKVFSNKLIGTFRMVLQKVVEESHVEVTDTLIDDNNAIIKTSLCVEVRYQA 120

:****:*************** *********.:*******:**.********.:******

Mouse_var1 TDGTVGPWDDGDFLGDESLQE-EKDSQETDGLLPGSRPSTRISGEKSFRSKGREKTKGGR 179

Human_var5 TDGTVGSWDDGDFLGDESLQEEEKDSQETDGLLPGSRPSSRPPGEKSFRR---------- 170

****** ************** *****************:* ******

Mouse_var1 DGEHKAGRSVFSAMKLGKTRSHKEEPQRQDEPAVLEMEDLDHLAIQLGDGLDPDSVSLAS 239

Human_var5 -----AGRSVFSAMKLGKNRSHKEEPQRPDEPAVLEMEDLDHLAIRLGDGLDPDSVSLAS 225

*************.********* ****************:**************

Mouse_var1 VTALTSNVSNKRSKPDIKMEPSAGRPMDYQVSITVIEARQLVGLNMDPVVCVEVGDDKKY 299

Human_var5 VTALTTNVSNKRSKPDIKMEPSAGRPMDYQVSITVIEARQLVGLNMDPVVCVEVGDDKKY 285

*****:******************************************************

Mouse_var1 TSMKESTNCPYYNEYFVFDFHVSPDVMFDKIIKISVIHSKNLLRSGTLVGSFKMDVGTVY 359

Human_var5 TSMKESTNCPYYNEYFVFDFHVSPDVMFDKIIKISVIHSKNLLRSGTLVGSFKMDVGTVY 345

************************************************************

Mouse_var1 SQPEHQFHHKWAILSDPDDISAGLKGYVKCDVAVVGKGDNIKTPHKANETDEDDIEGNLL 419

Human_var5 SQPEHQFHHKWAILSDPDDISSGLKGYVKCDVAVVGKGDNIKTPHKANETDEDDIEGNLL 405

*********************:**************************************

Mouse_var1 LPEGVPPERQWARFYVKIYRAEGLPRMNTSLMANVKKAFIGENKDLVDPYVQVFFAGQKG 479

Human_var5 LPEGVPPERQWARFYVKIYRAEGLPRMNTSLMANVKKAFIGENKDLVDPYVQVFFAGQKG 465

************************************************************

Mouse_var1 KTSVQKSSYEPLWNEQVVFTDLFPPLCKRMKVQIRDSDKVNDVAIGTHFIDLRKISNDGD 539

Human_var5 KTSVQKSSYEPLWNEQVVFTDLFPPLCKRMKVQIRDSDKVNDVAIGTHFIDLRKISNDGD 525

************************************************************

Mouse_var1 KGFLPTLGPAWVNMYGSTRNYTLLDEHQDLNEGLGEGVSFRARLMLGLAVEILDTSNPEL 599

Human_var5 KGFLPTLGPAWVNMYGSTRNYTLLDEHQDLNEGLGEGVSFRARLLLGLAVEIVDTSNPEL 585

********************************************:*******:*******

Mouse_var1 TSSTEVQVEQATPVSESCTGRMEEFFLFGAFLEASMIDRKNGDKPITFEVTIGNYGNEVD 659

Human_var5 TSSTEVQVEQATPISESCAGKMEEFFLFGAFLEASMIDRRNGDKPITFEVTIGNYGNEVD 645

*************:****:*:******************:********************

Mouse_var1 GMSRPLRPRPRKEPGDEEEVDLIQNSSDDEGDEAGDLASVSSTPPMRPQITDRNYFHLPY 719

Human_var5 GLSRPQRPRPRKEPGDEEEVDLIQNASDDEAGDAGDLASVSSTPPMRPQVTDRNYFHLPY 705

*:*** *******************:****..:****************:**********

Mouse_var1 LERKPCIYIKSWWPDQRRRLYNANIMDHIADKLEEGLNDVQEMIKTEKSYPERRLRGVLE 779

Human_var5 LERKPCIYIKSWWPDQRRRLYNANIMDHIADKLEEGLNDIQEMIKTEKSYPERRLRGVLE 765

***************************************:********************

Mouse_var1 ELSCGCHRFLSLSDKDQGRSSRTRLDRERLKSCMRELESMGQQAKSLRAQVKRHTVRDKL 839

Human_var5 ELSCGCCRFLSLADKDQGHSSRTRLDRERLKSCMRELENMGQQARMLRAQVKRHTVRDKL 825

****** *****:*****:*******************.*****: **************

Mouse_var1 RSCQNFLQKLRFLADEPQHSIPDVFIWMMSNNKRIAYARVPSKDLLFSIVEEELGKDCAK 899

Human_var5 RLCQNFLQKLRFLADEPQHSIPDIFIWMMSNNKRVAYARVPSKDLLFSIVEEETGKDCAK 885

* *********************:**********:****************** ******

Mouse_var1 VKTLFLKLPGKRGFGSAGWTVQAKLELYLWLGLSKQRKDFLCGLPCGFEEVKAAQGLGLH 959

Human_var5 VKTLFLKLPGKRGFGSAGWTVQAKVELYLWLGLSKQRKEFLCGLPCGFQEVKAAQGLGLH 945

************************:*************:*********:***********

Mouse_var1 SFPPISLVYTKKQAFQLRAHMYQARSLFAADSSGLSDPFARVFFINQSQCTEVLNETLCP 1019

Human_var5 AFPPVSLVYTKKQAFQLRAHMYQARSLFAADSSGLSDPFARVFFINQSQCTEVLNETLCP 1005

:***:*******************************************************

Mouse_var1 TWDQMLVFDNLELYGEAHELRDDPPIIVIEIYDQDSMGKADFMGRTFAKPLVKMADEAYC 1079

Human_var5 TWDQMLVFDNLELYGEAHELRDDPPIIVIEIYDQDSMGKADFMGRTFAKPLVKMADEAYC 1065

************************************************************

Mouse_var1 PPRFPPQLEYYQIYRGSATAGDLLAAFELLQIGPSGKADLPPINGPVDMDRGPIMPVPVG 1139

Human_var5 PPRFPPQLEYYQIYRGNATAGDLLAAFELLQIGPAGKADLPPINGPVDVDRGPIMPVPMG 1125

****************.*****************:*************:*********:*

Mouse_var1 IRPVLSKYRVEVLFWGLRDLKRVNLAQVDRPRVDIECAGKGVQSSLIHNYKKNPNFNTLV 1199

Human_var5 IRPVLSKYRVEVLFWGLRDLKRVNLAQVDRPRVDIECAGKGVQSSLIHNYKKNPNFNTLV 1185

************************************************************

Mouse_var1 KWFEVDLPENELLHPPLNIRVVDCRAFGRYTLVGSHAVSSLRRFIYRPPDRSAPNWNT-- 1257

Human_var5 KWFEVDLPENELLHPPLNIRVVDCRAFGRYTLVGSHAVSSLRRFIYRPPDRSAPSWNTTV 1245

******************************************************.***

Mouse_var1 ------------------TGEVVVSMEPEEPVKKLETMVKLDATSDAVVKVDVAEDEKER 1299

Human_var5 RLLRRCRVLCNGGSSSHSTGEVVVTMEPEVPIKKLETMVKLDATSEAVVKVDVAEEEKEK 1305

******:**** *:*************:*********:***:

Mouse_var1 KKKKKKGPSEEPEEEEPDESMLDWWSKYFASIDTMKEQLRQHETSGTDLEEKEEMESAEG 1359

Human_var5 K-KKKKGTAEEPEEEEPDESMLDWWSKYFASIDTMKEQLRQQEPSGIDLEEKEEVDNTEG 1364

* ***** :********************************:* ** *******::.:**

Mouse_var1 LKGPMKSKEKSRAAKEEKKKKNQSPGPGQGSEAPEKKKAKIDELKVYPKELESEFDSFED 1419

Human_var5 LKGSMKGKEKARAAKEEKKKKTQSSGSGQGSEAPEKKKPKIDELKVYPKELESEFDNFED 1424

*** **.***:**********.** * *********** *****************.***

Mouse_var1 WLHTFNLLRGKTGDDEDGSTEEERIVGRFKGSLCVYKVPLPEDVSREAGYDPTYGMFQGI 1479

Human_var5 WLHTFNLLRGKTGDDEDGSTEEERIVGRFKGSLCVYKVPLPEDVSREAGYDSTYGMFQGI 1484

*************************************************** ********

Mouse_var1 PSNDPINVLVRIYVVRATDLHPADINGKADPYIAIKLGKTDIRDKENYISKQLNPVFGKS 1539

Human_var5 PSNDPINVLVRVYVVRATDLHPADINGKADPYIAIRLGKTDIRDKENYISKQLNPVFGKS 1544

***********:***********************:************************

Mouse_var1 FDIEASFPMESMLTVAVYDWDLVGTDDLIGETKIDLENRFYSKHRATCGIAQTYSIHGYN 1599

Human_var5 FDIEASFPMESMLTVAVYDWDLVGTDDLIGETKIDLENRFYSKHRATCGIAQTYSTHGYN 1604

******************************************************* ****

Mouse_var1 IWRDPMKPSQILTRLCKEGKVDGPHFGPHGRVRVANRVFTGPSEIEDENGQRKPTDEHVA 1659

Human_var5 IWRDPMKPSQILTRLCKDGKVDGPHFGPPGRVKVANRVFTGPSEIEDENGQRKPTDEHVA 1664

*****************:********** ***:***************************

Mouse_var1 LSALRHWEDIPRVGCRLVPEHVETRPLLNPDKPGIEQGRLELWVDMFPMDMPAPGTPLDI 1719

Human_var5 LLALRHWEDIPRAGCRLVPEHVETRPLLNPDKPGIEQGRLELWVDMFPMDMPAPGTPLDI 1724

* **********.***********************************************

Mouse_var1 SPRKPKKYELRVIVWNTDEVVLEDDDFFTGEKSSDIFVRGWLKGQQEDKQDTDVHYHSLT 1779

Human_var5 SPRKPKKYELRVIIWNTDEVVLEDDDFFTGEKSSDIFVRGWLKGQQEDKQDTDVHYHSLT 1784

*************:**********************************************

Mouse_var1 GEGNFNWRYLFPFDYLAAEEKIVMSKKESMFSWDETEYKIPARLTLQIWDADHFSADDFL 1839

Human_var5 GEGNFNWRYLFPFDYLAAEEKIVISKKESMFSWDETEYKIPARLTLQIWDADHFSADDFL 1844

***********************:************************************

Mouse_var1 GAIELDLNRFPRGAKTAKQCTMEMATGEVDVPLVSIFKQKRVKGWWPLLARNENDEFELT 1899

Human_var5 GAIELDLNRFPRGAKTAKQCTMEMATGEVDVPLVSIFKQKRVKGWWPLLARNENDEFELT 1904

************************************************************

Mouse_var1 GKVEAELHLLTAEEAEKNPVGLARNEPDPLEKPNRPDTAFVWFLNPLKSIKYLICTRYKW 1959

Human_var5 GKVEAELHLLTAEEAEKNPVGLARNEPDPLEKPNRPDTAFVWFLNPLKSIKYLICTRYKW 1964

************************************************************

Mouse_var1 LIIKIVLALLGLLMLALFLYSLPGYMVKKLLGA 1992

Human_var5 LIIKIVLALLGLLMLGLFLYSLPGYMVKKLLGA 1997

***************.*****************

**A4.** Full length otoferlin var1 from 1 to 5979 (UniProt Q9ESF1-2, isoform 2). Translation 1992 a.a. MW=226101.60999999873. Protein translation of transcript variant 1 NCBI accession numbers NP_001093865.1 (top) and nucleotide sequence NM_001100395.1 (bottom) (5’-3’ orientation, primers in yellow (partly overlapping at fragment borders), start and stop codon for transcription bold/italics).

1 M A L I V H L K T V S E L R G K G D R I

1 ***atg***gccctgattgttcacctcaagactgtctcagagctccgaggcaaaggtgaccggatt -> fragment A

21 A K V T F R G Q S F Y S R V L E N C E G

61 gccaaagtcactttccgagggcagtctttctactcccgggtcctggagaactgcgagggt

41 V A D F D E T F R W P V A S S I D R N E

121 gtggctgactttgatgagacgttccggtggccagtggccagcagcatcgaccggaatgaa

61 V L E I Q I F N Y S K V F S N K L I G T

181 gtgttggagattcagattttcaactacagcaaagtcttcagcaacaagctgatagggacc

81 F C M V L Q K V V E E N R V E V T D T L

241 ttctgcatggtgctgcagaaagtggtggaggagaatcgggtagaggtgaccgacacgctg

101 M D D S N A I I K T S L S M E V R Y Q A

301 atggatgacagcaatgctatcatcaagaccagcctgagcatggaggtccggtatcaggcc

121 T D G T V G P W D D G D F L G D E S L Q

361 acagatggcactgtgggcccctgggatgatggagacttcctgggagatgaatccctccag

141 E E K D S Q E T D G L L P G S R P S T R

421 gaggagaaggacagccaggagacagatgggctgctacctggttcccgacccagcacccgg

161 I S G E K S F R S K G R E K T K G G R D

481 atatctggcgagaagagctttcgcagcaaaggcagagagaagaccaagggaggcagagat

181 G E H K A G R S V F S A M K L G K T R S

541 ggcgagcacaaagcgggaaggagtgtgttctcggccatgaaactcggcaaaactcggtcc

201 H K E E P Q R Q D E P A V L E M E D L D

601 cacaaagaggagccccaaagacaagatgagccagcagtgctggagatggaggacctggac

221 H L A I Q L G D G L D P D S V S L A S V

661 cacctagccattcagctgggggatgggctggatcctgactccgtgtctctagcctcggtc

241 T A L T S N V S N K R S K P D I K M E P

721 accgctctcaccagcaatgtctccaacaaacggtctaagccagatattaagatggagccc

261 S A G R P M D Y Q V S I T V I E A R Q L

781 agtgctggaaggcccatggattaccaggtcagcatcacagtgattgaggctcggcagctg

281 V G L N M D P V V C V E V G D D K K Y T

841 gtgggcttgaacatggaccctgtggtgtgtgtggaggtgggtgatgacaagaaatacacg

301 S M K E S T N C P Y Y N E Y F V F D F H

901 tcaatgaaggagtccacaaactgcccttactacaacgagtactttgtcttcgacttccat

321 V S P D V M F D K I I K I S V I H S K N

961 gtctctcctgatgtcatgtttgacaagatcatcaagatctcggttatccattctaagaac

341 L L R S G T L V G S F K M D V G T V Y S

1021 ctgcttcggagcggcaccctggtgggttccttcaaaatggatgtggggactgtgtattcc

361 Q P E H Q F H H K W A I L S D P D D I S

1081 cagcctgaacaccagttccatcacaaatgggccatcctgtcagaccccgatgacatctct

381 A G L K G Y V K C D V A V V G K G D N I

1141 gctgggttgaagggttatgtaaagtgtgatgtcgctgtggtgggcaagggagacaacatc

401 K T P H K A N E T D E D D I E G N L L L

1201 aagacaccccacaaggccaacgagacggatgaggacgacattgaagggaacttgctgctc

421 P E G V P P E R Q W A R F Y V K I Y R A

1261 cccgagggcgtgccccccgaacggcagtgggcacggttctatgtgaaaatttaccgagca

441 E G L P R M N T S L M A N V K K A F I G

1321 gagggactgccccggatgaacacaagcctcatggccaacgtgaagaaggcgttcatcggt

461 E N K D L V D P Y V Q V F F A G Q K G K

1381 gagaacaaggacctcgtcgacccctatgtgcaagtcttctttgctggacaaaagggcaaa

481 T S V Q K S S Y E P L W N E Q V V F T D

1441 acatcagtgcagaagagcagctatgagccgctatggaatgagcaggtcgtcttcacagac

501 L F P P L C K R M K V Q I R D S D K V N

1501 ttgttccccccactctgcaaacgcatgaaggtgcagatccgggactctgacaaggtcaat

521 D V A I G T H F I D L R K I S N D G D K

1561 gatgtggccatcggcacccacttcatcgacctgcgcaagatttccaacgatggagacaaa

541 G F L P T L G P A W V N M Y G S T R N Y

1621 ggcttcctgcctaccctcggtccagcctgggtgaacatgtacggctccacgcgcaactac

561 T L L D E H Q D L N E G L G E G V S F R

1681 acactgctggacgagcaccaggacttgaatgaaggcctgggggagggtgtgtccttccgg

581 A R L M L G L A V E I L D T S N P E L T

1741 gcccgcctcatgttgggactagctgtggagatcctggacacctccaacccagagctcacc

601 S S T E V Q V E Q A T P V S E S C T G R

1801 agctccacggaggtgcaggtggagcaggccacgcctgtctcggagagctgcacagggaga

621 M E E F F L F G A F L E A S M I D R K N

1861 atggaagaattttttctatttggagccttcttggaagcctcaatgattgaccggaaaaat

641 G D K P I T F E V T I G N Y G N E V D G

1921 ggggacaagccaattacctttgaggtgaccataggaaactacggcaatgaagtcgatggt

661 M S R P L R P R P R K E P G D E E E V D

1981 atgtcccggcccctgaggcctcggccccggaaagagcctggggatgaagaagaggtagac

681 L I Q N S S D D E G D E A G D L A S V S

2041 ctgattcagaactccagtgacgatgaaggtgacgaagccggggacctggcctcggtgtcc

701 S T P P M R P Q I T D R N Y F H L P Y L

2101 tccaccccacctatgcggccccagatcacggacaggaactatttccacctgccctacctg

721 E R K P C I Y I K S W W P D Q R R R L Y

2161 gagcgcaagccctgcatctatatcaagagctggtggcctgaccagaggcggcgcctctac

741 N A N I M D H I A D K L E E G L N D V Q

2221 aatgccaacatcatggatcacattgctgacaagctggaagaaggcctgaatgatgtacag

761 E M I K T E K S Y P E R R L R G V L E E

2281 gagatgatcaaaacggagaagtcctacccggagcgccgcctgcggggtgtgctagaggaa

781 L S C G C H R F L S L S D K D Q G R S S

2341 ctcagctgtggctgccaccgcttcctctccctctcggacaaggaccagggccgctcgtcc

801 R T R L D R E R L K S C M R E L E S M G

2401 cgcaccaggctggatcgagagcgtcttaagtcctgtatgagggagttggagagcatggga

821 Q Q A K S L R A Q V K R H T V R D K L R

2461 cagcaggccaagagcctgagggctcaggtgaagcggcacactgttcgggacaagctgagg

841 S C Q N F L Q K L R F L A D E P Q H S I

2521 tcatgccagaactttctgcagaagctacgcttcctggcggatgagccccagcacagcatt

861 P D V F I W M M S N N K R I A Y A R V P

2581 cctgatgtgttcatttggatgatgagcaacaacaaacgtatcgcctatgcccgcgtgcct fragment B ->

881 S K D L L F S I V E E E L G K D C A K V

2641 tccaaagacctgctcttctccatcgtggaggaggaactgggcaaggactgcgccaaagtc

901 K T L F L K L P G K R G F G S A G W T V

2701 aagaccctcttcctgaagctgccagggaagaggggcttcggctcggcaggctggacagta

921 Q A K L E L Y L W L G L S K Q R K D F L

2761 caggccaagctggagctctacctgtggctgggcctcagcaagcagcgaaaggacttcctg

941 C G L P C G F E E V K A A Q G L G L H S

2821 tgtggtctgccctgtggcttcgaggaggtcaaggcagcccaaggcctgggcctgcattcc

961 F P P I S L V Y T K K Q A F Q L R A H M

2881 tttccgcccatcagcctagtctacaccaagaagcaagccttccagctccgagcacacatg

981 Y Q A R S L F A A D S S G L S D P F A R

2941 tatcaggcccgaagcctctttgctgctgacagcagtgggctctctgatccctttgcccgt

1001 V F F I N Q S Q C T E V L N E T L C P T

3001 gtcttcttcatcaaccagagccaatgcactgaggttctaaacgagacactgtgtcccacc

1021 W D Q M L V F D N L E L Y G E A H E L R

3061 tgggaccagatgctggtatttgacaacctggagctgtacggtgaagctcacgagttacga

1041 D D P P I I V I E I Y D Q D S M G K A D

3121 gatgatccccccatcattgtcattgaaatctacgaccaggacagcatgggcaaagccgac

1061 F M G R T F A K P L V K M A D E A Y C P

3181 ttcatgggccggaccttcgccaagcccctggtgaagatggcagatgaagcatactgccca

1081 P R F P P Q L E Y Y Q I Y R G S A T A G

3241 cctcgcttcccgccgcagcttgagtactaccagatctaccgaggcagtgccactgccgga

1101 D L L A A F E L L Q I G P S G K A D L P

3301 gacctactggctgccttcgagctgctgcagattgggccatcagggaaggctgacctgcca

1121 P I N G P V D M D R G P I M P V P V G I

3361 cccatcaatggcccagtggacatggacagagggcccatcatgcctgtgcccgtgggaatc

1141 R P V L S K Y R V E V L F W G L R D L K

3421 cggccagtgctcagcaagtaccgagtggaggtgctgttctggggcctgagggacctaaag

1161 R V N L A Q V D R P R V D I E C A G K G

3481 agggtgaacctggcccaggtggaccgaccacgggtggacatcgagtgtgcaggaaagggg

1181 V Q S S L I H N Y K K N P N F N T L V K

3541 gtacaatcctccctgattcacaattataagaagaaccccaacttcaacacgctggtcaag

1201 W F E V D L P E N E L L H P P L N I R V

3601 tggtttgaagtggacctcccggagaatgagctcctgcacccacccttgaacatccgagtg

1221 V D C R A F G R Y T L V G S H A V S S L

3661 gtagattgccgggcctttggacgatacaccctggtgggttcccacgcagtcagctcactg

1241 R R F I Y R P P D R S A P N W N T T G E

3721 aggcgcttcatctaccgacctccagaccgctcagcccccaactggaacaccacaggggag

1261 V V V S M E P E E P V K K L E T M V K L

3781 gttgtagtaagcatggagcctgaggagccagttaagaagctggagaccatggtgaaactg

1281 D A T S D A V V K V D V A E D E K E R K

3841 gatgcgacttctgatgctgtggtcaaggtggatgtggctgaagatgagaaggaaaggaag

1301 K K K K K G P S E E P E E E E P D E S M

3901 aagaagaaaaagaaaggcccgtcagaggagccagaggaggaagagcccgatgagagcatg

1321 L D W W S K Y F A S I D T M K E Q L R Q

3961 ctggattggtggtccaagtacttcgcctccatcgacacaatgaaggagcaacttcgacaa

1341 H E T S G T D L E E K E E M E S A E G L

4021 catgagacctctggaactgacttggaagagaaggaagagatggaaagcgctgagggcctg

1361 K G P M K S K E K S R A A K E E K K K K

4081 aagggaccaatgaagagcaaggagaagtccagagctgcaaaggaggagaaaaagaagaaa

1381 N Q S P G P G Q G S E A P E K K K A K I

4141 aaccagagccctggccctggccagggatcggaggctcctgagaagaagaaagccaagatc

1401 D E L K V Y P K E L E S E F D S F E D W

4201 gatgagcttaaggtgtaccccaaggagctggaatcggagtttgacagctttgaggactgg

1421 L H T F N L L R G K T G D D E D G S T E

4261 ctgcacaccttcaacctgttgaggggcaagacgggagatgatgaggatggctccacagag

1441 E E R I V G R F K G S L C V Y K V P L P

4321 gaggagcgcatagtaggccgattcaagggctccctctgtgtgtacaaagtgccactccca

1461 E D V S R E A G Y D P T Y G M F Q G I P

4381 gaagatgtatctcgagaagctggctatgatcccacctatggaatgttccagggcatccca

1481 S N D P I N V L V R I Y V V R A T D L H

4441 agcaatgaccccatcaatgtgctggtccgaatctatgtggtccgggccacagacctgcac

1501 P A D I N G K A D P Y I A I K L G K T D

4501 ccggccgacatcaatggcaaagctgacccctatattgccatcaagttaggcaagaccgac

1521 I R D K E N Y I S K Q L N P V F G K S F

4561 atccgagacaaggagaactacatctccaagcagctcaaccctgtgtttgggaagtccttt

1541 D I E A S F P M E S M L T V A V Y D W D

4621 gacattgaggcctccttccccatggagtccatgttgacagtggccgtgtacgactgggat

1561 L V G T D D L I G E T K I D L E N R F Y

4681 ctggtgggcactgatgacctcatcggagaaaccaagattgacctggaaaaccgcttctac

1581 S K H R A T C G I A Q T Y S I H G Y N I

4741 agcaagcatcgcgccacctgcggcatcgcacagacctattccatacatggctacaatatc

1601 W R D P M K P S Q I L T R L C K E G K V

4801 tggagggaccccatgaagcccagccagatcctgacacgcctctgtaaagagggcaaagtg

1621 D G P H F G P H G R V R V A N R V F T G

4861 gacggcccccactttggtccccatgggagagtgagggttgccaaccgtgtcttcacgggg

1641 P S E I E D E N G Q R K P T D E H V A L

4921 ccttcagaaatagaggatgagaatggtcagaggaagcccacagatgagcacgtggcactg

1661 S A L R H W E D I P R V G C R L V P E H

4981 tctgctctgagacactgggaggacatcccccgggtgggctgccgccttgtgccggaacac

1681 V E T R P L L N P D K P G I E Q G R L E

5041 gtggagaccaggccgctgctcaaccctgacaagccaggcattgagcagggccgcctggag

1701 L W V D M F P M D M P A P G T P L D I S

5101 ctgtgggtggacatgttccccatggacatgccagcccctgggacacctctggatatatcc

1721 P R K P K K Y E L R V I V W N T D E V V

5161 cccaggaaacccaagaagtacgagctgcgggtcatcgtgtggaacacagacgaggtggtc fragment C ->

1741 L E D D D F F T G E K S S D I F V R G W

5221 ctggaagacgatgatttcttcacgggagagaagtccagtgacatttttgtgagggggtgg

1761 L K G Q Q E D K Q D T D V H Y H S L T G

5281 ctgaagggccagcaggaggacaaacaggacacagatgtccactatcactccctcacgggg

1781 E G N F N W R Y L F P F D Y L A A E E K

5341 gagggcaacttcaactggagatacctcttccccttcgactacctagcggccgaagagaag

1801 I V M S K K E S M F S W D E T E Y K I P

5401 atcgttatgtccaaaaaggagtctatgttctcctgggatgagacggagtacaagatccct

1821 A R L T L Q I W D A D H F S A D D F L G

5461 gcgcggctcaccctgcagatctgggacgctgaccacttctcggctgacgacttcctgggg

1841 A I E L D L N R F P R G A K T A K Q C T

5521 gctatcgagctggacctgaaccggttcccgaggggcgctaagacagccaagcagtgcacc

1861 M E M A T G E V D V P L V S I F K Q K R

5581 atggagatggccaccggggaggtggacgtacccctggtttccatctttaaacagaaacgt

1881 V K G W W P L L A R N E N D E F E L T G

5641 gtcaaaggctggtggcccctcctggcccgcaatgagaatgatgagtttgagctcacaggc

1901 K V E A E L H L L T A E E A E K N P V G

5701 aaagtggaggcggagctacacctactcacggcagaggaggcagagaagaaccctgtgggc

1921 L A R N E P D P L E K P N R P D T A F V

5761 ctggctcgcaatgaacctgatcccctagaaaaacccaaccggcctgacacggcattcgtc

1941 W F L N P L K S I K Y L I C T R Y K W L

5821 tggttcctgaacccactcaaatctatcaagtacctcatctgcacccggtacaagtggctg

1961 I I K I V L A L L G L L M L A L F L Y S

5881 atcatcaagatcgtgctggcgctgctggggctgctcatgctggccctcttcctttacagc

1981 L P G Y M V K K L L G A *

5941 ctcccaggctacatggtcaagaagctcctaggggcc***taa***
