# Supplementary material for: Overloaded Adeno-Associated Virus as a Novel Gene Therapeutic Tool for Otoferlin-Related Deafness
Source: Front Mol Neurosci. 2021 Jan 7;13:600051. doi: 10.3389/fnmol.2020.600051 (PMC7817888; doi:10.3389/fnmol.2020.600051)
Supplement: Supplementary file 2 [file Table_2.docx]

Overloaded adeno-associated virus as a novel gene therapeutic tool for otoferlin-related deafness

Vladan Rankovic^1,2,#,*^, Christian Vogl^1,3,4,#,*^, Nele M. Dörje^1,3^, Iman Bahader^4,5^, Carlos J. Duque-Afonso^1,6,7^, Anupriya Thirumalai^1^, Thomas Weber^1^, Kathrin Kusch^1^, Nicola Strenzke^4,5^, and Tobias Moser^1,4,6,7,8,9*^

**Supplemental material:**

**Supp. Table 1. AAV serotypes used in the study with obtained titers, purity and ABR rescue efficacy (gc, genome copies).**


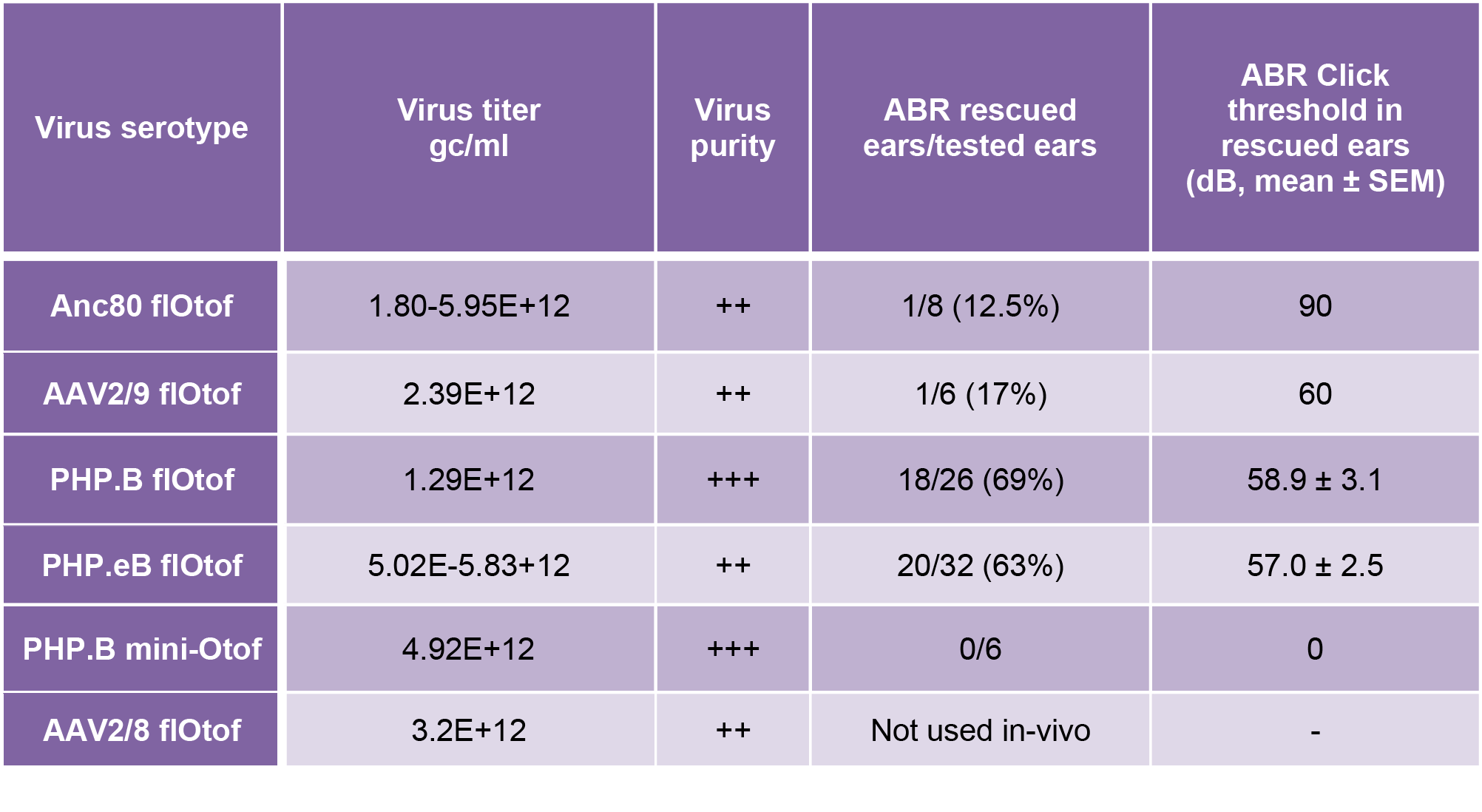


**
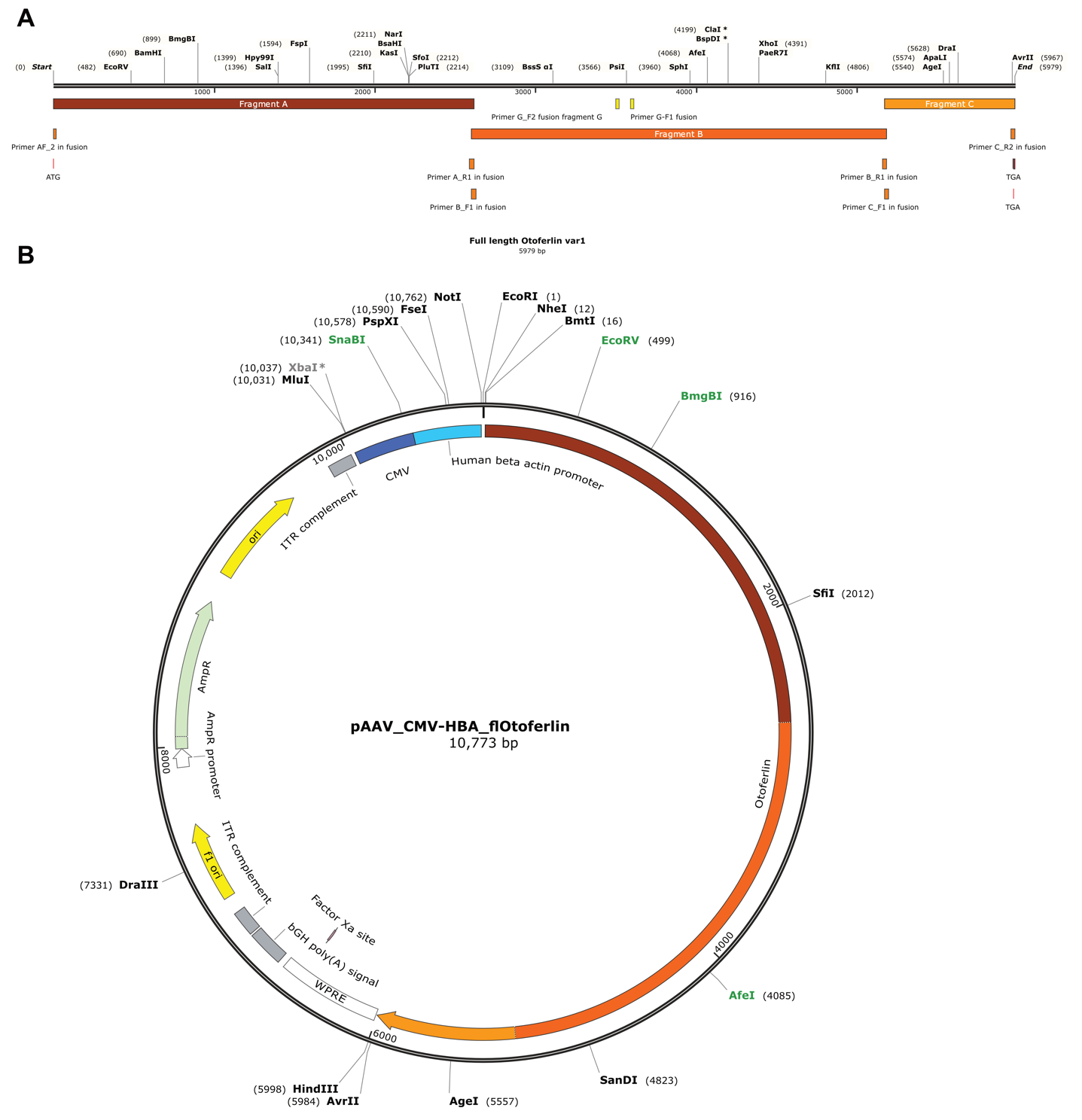
**

**Supp. Figure 1:** ***fl-Otof* construct assembly and pAAV vector used for AAV packaging of *fl-Otof*.** (**A**) linearized otoferlin sequence including primer binding sets used for the construct assembly. (**B**) pAAV cis-plasmid providing fl-otoferlin. Color code of *fl-Otof*-CDS corresponds to the fragments shown in panel A.

**
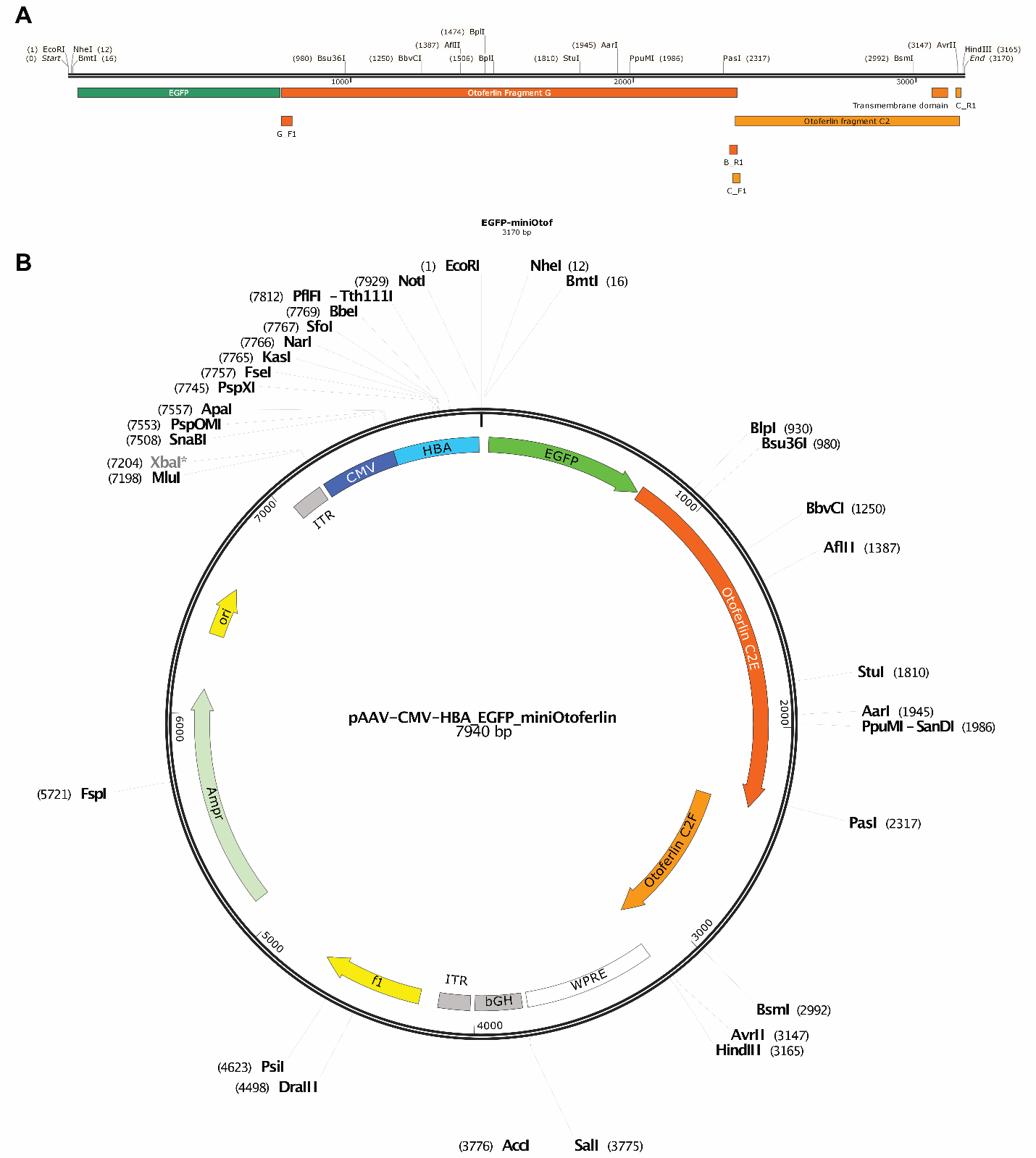
**

**Supp. Figure 2:** ***mini-Otof* construct assembly and pAAV vector used for AAV packaging.** (**A**) linearized mini-otoferlin sequence including primer binding sets used for the construct assembly. (**B**) pAAV cis-plasmid providing fused protein of EGFP and mini-Otof (C2E and C2F including the transmembrane domain).

**
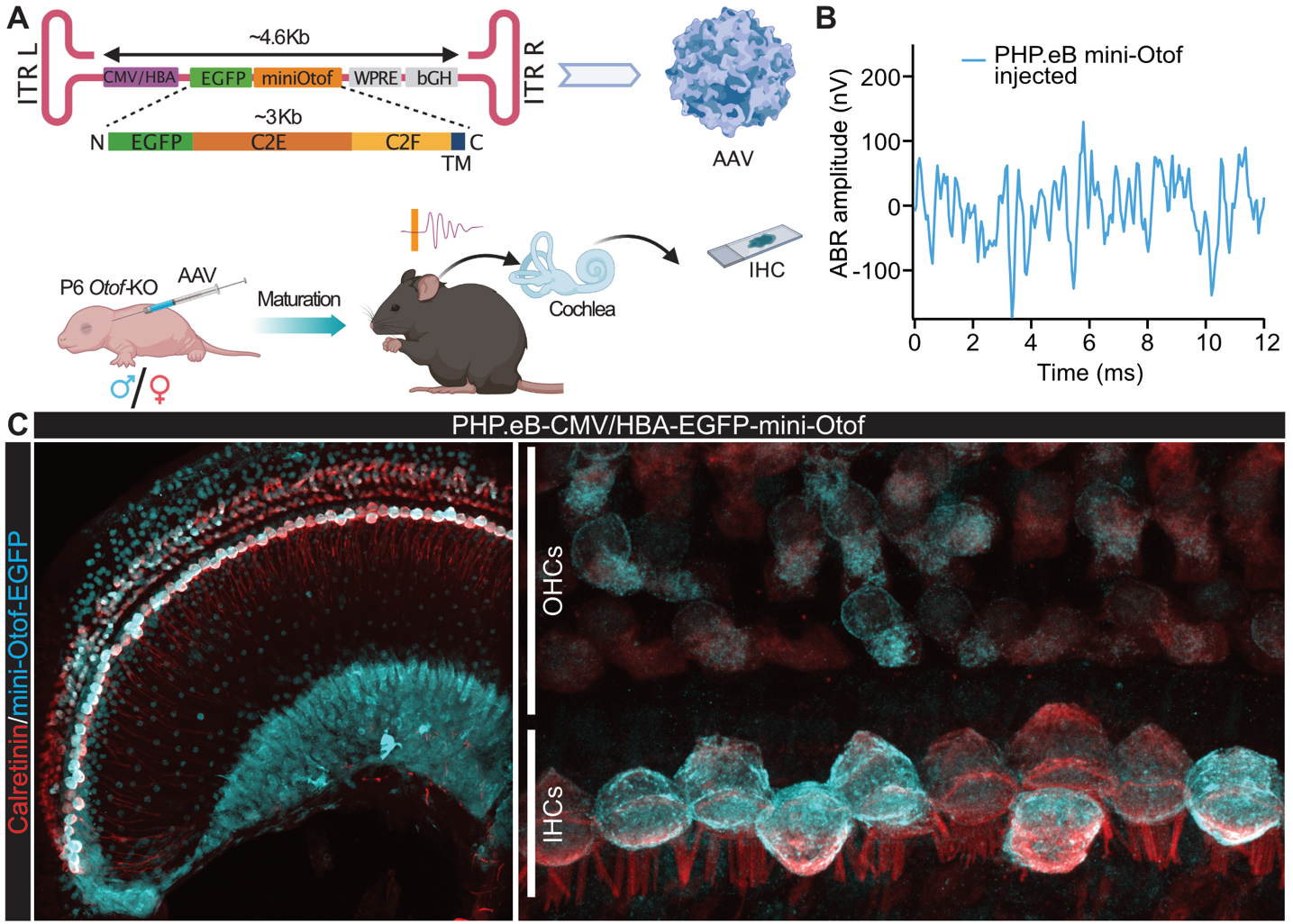
**

**Supp. Figure 3: Insert size appears to determine AAV transduction/otoferlin expression rate.** (**A**) Schematic illustration of the mini-Otof AAV construct design and experimental approach. The AAV/promoter construct is identical to the one used in our other experiments, but only encodes a short C-terminal and GFP-tagged otoferlin fragment (*‘mini-Otof’*) instead of the *fl-Otof*-CDS. (**B**) Treatment with the ‘mini-Otof’ AAV virus does not rescue hearing function assessed by ABRs to 100dB click stimulation despite (**C**) abundant viral transfection of IHC and OHC in the same ear (confocal z-projection, representative example).


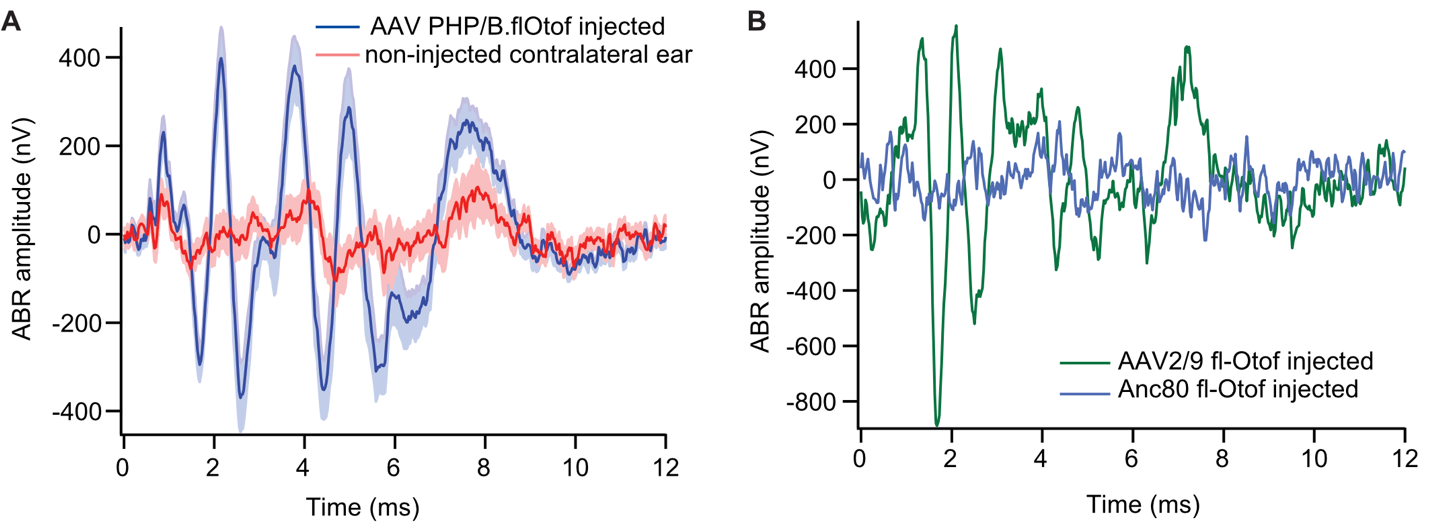


**Supp. Figure 4: Rescue of hearing function in *Otof*-KO mice: ABR after successful rescue with other Otof viruses** (related to Fig. 2 and suppl. Table 1). **(A)** Grand averages of ABR waveforms to 100 dB Click stimulation in *Otof*-KO mice with successful partial rescue of hearing function by PHP.B *fl-Otof* AAV (blue, n=14). ABRs from non-injected contralateral ears of the same animals are shown in red. (**B**) ABR waveforms to 100 dB click stimulation from the single rescued ears injected with Anc80 (blue) and AAV2/9 (green) viruses.
